# Recreational cannabis legalization has had limited effects on a wide range of adult psychiatric and psychosocial outcomes

**DOI:** 10.1017/S0033291722003762

**Published:** 2023-10

**Authors:** Stephanie M. Zellers, J. Megan Ross, Gretchen R. B. Saunders, Jarrod M. Ellingson, Tasha Walvig, Jacob E. Anderson, Robin P. Corley, William Iacono, John K. Hewitt, Christian J. Hopfer, Matt K. McGue, Scott Vrieze

**Affiliations:** 1 Department of Psychology, University of Minnesota, Minneapolis, Minnesota, USA; 2 Institute for Molecular Medicine Finland, University of Helsinki, Helsinki, Finland; 3 Department of Psychiatry, University of Colorado Anschutz Medical Campus, Aurora, Colorado, USA; 4 Institute for Behavioral Genetics, University of Colorado Boulder, Boulder, Colorado, USA; 5 Department of Psychology and Neuroscience, University of Colorado Boulder, Boulder, Colorado, USA

**Keywords:** Co-twin control, discordant twin, drug abuse, longitudinal, psychopathology, substance use disorder

## Abstract

**Background:**

The causal impacts of recreational cannabis legalization are not well understood due to the number of potential confounds. We sought to quantify possible causal effects of recreational cannabis legalization on substance use, substance use disorder, and psychosocial functioning, and whether vulnerable individuals are more susceptible to the effects of cannabis legalization than others.

**Methods:**

We used a longitudinal, co-twin control design in 4043 twins (*N* = 240 pairs discordant on residence), first assessed in adolescence and now age 24–49, currently residing in states with different cannabis policies (40% resided in a recreationally legal state). We tested the effect of legalization on outcomes of interest and whether legalization interacts with established vulnerability factors (age, sex, or externalizing psychopathology).

**Results:**

In the co-twin control design accounting for earlier cannabis frequency and alcohol use disorder (AUD) symptoms respectively, the twin living in a recreational state used cannabis on average more often (*β*_w_ = 0.11, *p* = 1.3 × 10^−3^), and had fewer AUD symptoms (*β*_w_ = −0.11, *p* = 6.7 × 10^−3^) than their co-twin living in an non-recreational state. Cannabis legalization was associated with no other adverse outcome in the co-twin design, including cannabis use disorder. No risk factor significantly interacted with legalization status to predict any outcome.

**Conclusions:**

Recreational legalization was associated with increased cannabis use and decreased AUD symptoms but was not associated with other maladaptations. These effects were maintained within twin pairs discordant for residence. Moreover, vulnerabilities to cannabis use were not exacerbated by the legal cannabis environment. Future research may investigate causal links between cannabis consumption and outcomes.

Cannabis has been illegal in the United States at the federal level since 1970, but as of January 2022, nearly half the US population lived in a state where cannabis has been legalized for recreational consumption, despite the potential negative effects. Approximately 30% of cannabis users develop a cannabis use disorder, and that risk of disorder increases with greater cannabis use (CDC, n.d.). Cannabis use is also associated with problems in other major domains of human behavior: other substance use, psychopathology, cognitive ability, motivation, employment, and interpersonal relationships (Brook, Lee, Finch, Seltzer, & Brook, 2013; CDC, n.d.; Richmond-Rakerd, Slutske, & Wood, 2017; Volkow, Baler, Compton, & Weiss, 2014; Volkow et al., 2016; Zellers, Iacono, McGue, & Vrieze, 2022).

As cannabis becomes readily available due to recreational legalization, it follows that cannabis use will become more prevalent. Indeed, studies of recreational legalization have found increases in prevalence of cannabis use in adults (Cerdá et al., 2020; Parnes, Smith, & Conner, 2018; Steigerwald et al., 2020; Wallace et al., 2020). A related concern then, is to what degree increases in prevalence of cannabis use will be accompanied by increases in negative outcomes associated with cannabis intake.

Supporters and opponents of recreational cannabis legalization often cite possible outcomes of legalization as their rationale for or against recreational policies; supporters typically cite potential benefits or reduced harms as compared to other substances, whereas opponents typically cite harms to society and addictive potential (Jones, 2019; Pew Research Center, 2015). Cannabis is an addictive substance and recreational cannabis legalization has been associated with increased prevalence of cannabis use disorder (Cerdá et al., 2020) and increased use of high-potency products (Hasin et al., 2021). The effects of legalization on other outcomes, be it benefits or harms, are less well-studied.

Anderson and Rees (2021) comprehensively review the legalization research on outcomes other than cannabis intake and use disorder. In short, recreational legalization may be associated with decreases in alcohol and opiate consumption, as well as decreases in the physical health consequences associated with their consumption. The authors speculate that decreases in substance consumption could drive reductions in mental health concerns, but current evidence is not conclusive. To our knowledge, no studies have investigated the impacts of recreational legalization on other indicators of psychiatric and psychosocial functioning.

It remains difficult to know whether recreational legalization causes changes in substance use, psychiatric outcomes, and psychosocial functioning due to the number of potential confounders that must be addressed. Quasi-experimental designs can extend the existing research to investigate the potentially causal effects of recreational legalization on psychiatric and psychosocial outcomes.

In addition, it remains uncertain whether some individuals may be disproportionately affected by recreational policies, an important consideration in prevention and intervention efforts (Cerdá et al., 2020). There is mixed evidence for differential vulnerability by age to the impacts of recreational legalization on prevalence and frequency of cannabis use (Bae & Kerr, 2019; Cerdá et al., 2020; Kerr, Bae, Phibbs, & Kern, 2017; Parnes et al., 2018). Additionally, men use cannabis more frequently than women, have greater rates of problematic use, and may be more sensitive to permissive social environments (Brown et al., 2008; O'Malley & Johnston, 2002), though there is some evidence to suggest a stronger effect of recreational legalization on prevalence and frequency of use in women as compared to men (Bae & Kerr, 2019). Lastly, externalizing psychopathology confers risk for cannabis use, which in turn exacerbates existing psychopathology (Elkins et al., 2018; Iacono, Malone, & McGue, 2008). To our knowledge, externalizing psychopathology has not yet been investigated with respect to vulnerability to legalization effects (Anderson & Rees, 2021; Hasin & Aharonovich, 2020).

We expand on the literature in two ways. First, we employ a quasi-experimental study, including a co-twin control analysis, examining twin pairs discordant for living in a state with legal recreational cannabis, to rigorously examine the potential causal effect of cannabis legalization on a broad range of psychiatric and psychosocial outcomes. Our design accounts for within-individual change and secular trends, and implicitly controls for factors contributing to these outcomes, such as genes, age, sex, and rearing environment. In other words, our co-twin control design evaluates whether the twin living in a recreationally legal state has worse outcomes than their twin in an illegal state, even though twins share important aspects of their upbringing (ex. social norms around substance use, parental permissiveness, etc.) as well as some or all of their genes that influence substance use and related outcomes (completely shared by monozygotic twins, 50% shared by dizygotic twins). Second, we investigate prospectively assessed risk factors and their relationship to cannabis use and other outcomes in recreationally legal and illegal environments. Our study design and hypotheses were pre-registered on 28 December 2021, available at https://osf.io/km3f7/.

## Methods

### Participants

We analyzed data from *N* = 4078 individuals drawn from longitudinal community twin samples maintained by the Minnesota Center for Twin Family Research (Wilson et al., 2019), and the Colorado Center for Antisocial Drug Dependence (Corley, Reynolds, Wadsworth, Rhea, & Hewitt, 2019). Participants were recruited in adolescence via birth records from the years 1972–1994; additional assessment details were described previously (Corley et al., 2019; Wilson et al., 2019). Prior to the first recreational dispensaries opening in Colorado in 2014, individuals in each sample had been independently studied. After this point, investigators from both sites conducted a joint assessment of these same twins. All participants provided informed consent at each assessment; parents provided informed consent when participants were minors.

### Measures

#### Residence exposure

Using an established definition of recreational legalization based on date of passage of recreational policies (RAND-USC Schaeffer Opioid Policy Tools and Information Center, 2021), participants were classified as living in a recreationally legal or illegal state based on ZIP code and date of assessment. We also conducted sensitivity checks for this decision, described in the supplement. Those without a valid US ZIP code were excluded (*N* = 35) resulting in an analytic sample of *N* = 4043 individuals.

#### Pre-2014 covariates and indicators of vulnerability

Full harmonization details for each measure are presented in the supplement and online Supplementary Tables S1–S2. Frequency of cannabis, alcohol, and tobacco consumption were measured as ‘number of days used in the last 180’. Use disorder was operationalized as symptom counts for cannabis, alcohol, and nicotine experienced in the participants' lifetime prior to 2014. We also measured parental education at intake, symptoms of adolescent non-substance related externalizing psychopathology (conduct disorder and ADHD (Zellers et al., 2020)), and lifetime symptoms of ASPD experienced prior to 2014. The two measures of externalizing are intended to approximate risk both prior to and after the initiation of substance use.

#### Post-2014 drug, psychiatric, and psychosocial outcomes

The supplement contains full descriptions of each outcome. Consumption of alcohol, tobacco, and cannabis was assessed as ‘number of days used in the last 180’. We also measured number of non-cannabis illicit substances consumed. Psychiatric outcomes included DSM-5 alcohol, tobacco, and cannabis use disorder symptom counts, and disordered personality traits assessed at the domain level: negative affectivity, detachment, psychoticism, and disinhibition (Krueger, Derringer, Markon, Watson, & Skodol, 2012). Psychosocial outcomes included the following adapted scales: Externalizing Spectrum Inventory (Patrick, Kramer, Krueger, & Markon, 2013), Dyadic Adjustment Scale (Spanier & Thompson, 1982), Occupational Citizenship and Counterproductive Work Behavior checklists (Spector, Bauer, & Fox, 2010), Civic Engagement Scale (Doolittle & Faul, 2013), and the International Cognitive Ability Resource (Condon & Revelle, 2014). We also assessed several psychosocial outcomes through scales created for this assessment based on the older independent assessments at each site; scales and example items are as follows: savings habits (ex. I have a retirement plan), financial problems (ex. I defaulted on a credit card payment), degree of unemployment (ex. my hours were reduced), and legal consequences (ex. I have been to court).

### Analyses

#### Population-Level effects of recreational legalization

We tested the effects of living in a recreational cannabis state with an individual-level mixed effects model accounting for family, where 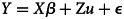. In this equation, the outcome *Y* is a function of *X*, the design matrix that includes fixed effects, fixed-effects coefficient vector *β*, *Z* vector of random effects for individual nested within family *u*, and 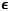, the error term. The individual level model does not leverage the additional information provided by data on twin pairs, instead it is an analysis comparable to that of unrelated individuals, but includes the vector *Z* of random effects to account for non-independence of data when analytically treating twins as individuals rather than as pairs.

All models included covariates of age, sex, and intake cohort, defined as being part of the cohort selected for higher childhood externalizing symptoms as compared to the non-selected community cohorts (Keyes et al., 2009). When comparable measures were available longitudinally, the pre-2014 measure was included as a covariate. For example, when post-2014 cannabis frequency was the outcome, pre-2014 cannabis frequency was included as a covariate. The supplement includes an exhaustive list of models and included covariates. All outcome variables were transformed to Z-scores to facilitate interpretation of difference in standard deviation units of outcome variable as a result of moving from 0 to 1 on the dummy-coded residence predictor. Consumption frequency variables were log-transformed.

#### Co-twin control analyses

We followed up all individual level models with co-twin control models, both pooled MZ-DZ and zygosity-stratified, to better account for unmeasured confounders shared within a twin pair. These unmeasured confounders comprise other factors of interest, such as genes, rearing environment and parental education, to the extent that those factor make twins similar on a given outcome. The co-twin control model evaluates whether twins living in recreational states are different on each outcome as compared to their co-twin living in non-recreational states. If within-pair differences exist for an outcome, and the effect is comparable in magnitude to the individual level effect, this is consistent with a causal effect of recreational legalization on that outcome (McGue, Osler, & Christensen, 2010). On the other hand, if there is an individual level effect but there are no within-pair differences, this is consistent with an effect of legalization due to genetic and/or environmental confounders.

The co-twin mixed effects model decomposed the effect of legalization into within-twin-pair and between-twin-pair effects as follows: 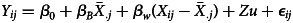. Here Y is a function of the average exposure for a given twin-pair (between-pair effect *β*_*B*_) and the effect of residence discordance of each twin from their cotwin (within-pair effect *β*_*w*_). Arguably, this analysis restricted to monozygotic twins offers the estimate of *β*_*w*_ least affected by unmeasured confounds, as monozygotic twins share all of their genes (McGue et al., 2010; Saunders, Mcgue, & Malone, 2019). To formally compare *B*_*W*_ to *B*, we computed the mean difference between standardized *B*_*W*_ and *B* and the 95% confidence interval around the difference across 1000 bootstrap replicates.

#### Attrition and sensitivity analyses

Attrition was assessed with a mixed effects model, regressing drop-out on each pre-legalization covariate, as well as sex, and also addressing non-independence of twin data. We also evaluated site-specific attrition by evaluating whether dropout likelihood varied by site for each pre-legalization covariate. Attrition results were then utilized in a sensitivity analysis. This attrition analysis, and other sensitivity analyses are described in detail in the supplement. We evaluated a stricter definition of recreational policy as well as alternative definitions of the substance consumption and dependence phenotypes. We also evaluated robustness of our findings to the longitudinal covariates and effects of Covid-19 on financial outcomes.

#### Differential vulnerability

We tested the extent to which some individuals may be more vulnerable to the effects of recreational legalization than others. We evaluated the risk factors of age, sex, adolescent externalizing psychopathology, and adult ASPD. We used mixed effects models to evaluate three effects of interest: recreational legalization, the risk factor, and their interaction. A significant interaction term was interpreted as evidence of differential vulnerability. Posthoc interaction power analyses were conducted via the method described by Baranger et al. (2022) using the observed correlations between predictors, the outcome, and the interaction term.

#### Statistical software

Data cleaning, analyses, and plotting were conducted in RStudio using lme4 (Bates, Mächler, Bolker, & Walker, 2015), lmerTest (Kuznetsova, Brockhoff, & Christensen, 2017), and ggplot2 (Wickham, 2016). Given the large number of related analyses, we corrected for multiple non-independent tests by determining number of effective tests for correlated variables and calculating alpha levels that maintained a family-wise error rate of 0.05 (Derringer, 2018). We arrived at an adjusted *p* value threshold of *α* = 1.7 × 10^−3^ for descriptive comparisons, *α* = 2.3 × 10^−3^ for the individual-level and co-twin analyses, and *α* = 4.6 × 10^−4^ for differential vulnerability analyses.

## Results

### Descriptives

The analytic sample (*N* = 4043) was 57.5% female and 53.0% monozygotic pairs. Consistent with the birth cohorts and states from which these participants were drawn, 92% of participants were White and 5% of participants reported Hispanic ethnicity. At the assessment at which outcomes were assessed, participants ranged from ages 24–49 (*M* = 35.0, s.d. = 5.1) and dates of assessment ranged between 2018 and 2021. For pre-2014 measures of alcohol, tobacco, and cannabis frequency, individuals ranged from ages 16–34 (*M* = 24.8, s.d. = 3.7) and assessment dates ranged from 1996–2013, though the majority of assessments (81%) occurred between 2009–2013 (see online Supplementary Fig. S1 for a depiction of ages and assessment years).

In terms of residence, 40% of individuals resided in recreationally legal states at the time of assessment, the remaining 60% lived in states without recreational cannabis. Most twin pairs were concordant for residence, but 240 twin pairs were discordant for residence, meaning that one twin lived in a recreationally legal state whereas their co-twin lived in an non-recreational state. We defined recreational legalization as a binary variable in our analyses and did not differentiate between comprehensive medical (THC and CBD), limited medical (CBD only), and no medical policy states because 80% of our participants living in non-recreational states (total *N* = 2346) lived in states with comprehensive medical policies, as compared to those living in limited medical (*N* = 368) and no medical policy (*N* = 78) states. Very few participants (*N* = 72) reported having medical cannabis cards; of these 72 individuals, 23 resided in non-recreational states, 48 resided in recreational states, and one resided internationally.

Table 1 presents means, standard deviations, and ranges for outcomes in the main text, and online Supplementary Table S3 presents descriptives for outcomes for the sensitivity analyses. Most phenotypes did not differ in simple means between recreational and non-recreational groups, though there were some differences for pre-2014 variables; these pre-legalization differences may reflect differences by recruitment site or trends prior to legalization. Individual level correlations are presented in online Supplementary Fig. S2 and twin correlations are presented in online Supplementary Table S4. Twin correlations were generally significantly positive and larger in monozygotic twins as compared to dizygotic twins, a rough index of moderate heritability of all variables.

**Table 1.** Descriptives for continuous outcomes and covariates

Phenotype	Full sample mean	Full sample s.d.	Rec. mean	Rec. s.d.	Non-rec. mean	Non-rec. s.d.
Pre-2014 covariates						
Cannabis frequency*	11.5	38.1	13.3	40.9	10.2	36.0
Alcohol frequency*	**25.4**	**33.1**	**26.8**	**34.7**	**24.1**	**31.3**
Nicotine frequency*	**42.7**	**71.8**	**35.6**	**67.6**	**47.5**	**74.1**
Cannabis use disorder symptoms	0.8	1.8	0.9	1.8	0.7	1.7
Alcohol use disorder symptoms	**1.6**	**2.2**	**2.0**	**2.3**	**1.4**	**2.1**
Nicotine dependence symptoms	1.2	1.8	1.2	1.8	1.1	1.8
Adolescent externalizing symptoms	1.6	2.4	1.7	2.5	1.5	2.3
ASPD Symptoms	1.3	1.6	1.6	1.7	1.1	1.4
Current outcomes						
Cannabis frequency*	**15.9**	**44.9**	**19.5**	**49.3**	**13.6**	**41.7**
Alcohol frequency*	39.7	47.9	38.8	47.4	40.2	48.1
Nicotine frequency*	29.4	62.8	27.5	61.1	30.7	64.0
Other illicit drug use	0.2	0.6	0.2	0.7	0.1	0.6
Cannabis use disorder symptoms	0.2	0.9	0.2	0.8	0.2	0.9
Alcohol use disorder symptoms	0.5	1.3	0.4	1.3	0.5	1.3
Nicotine dependence symptoms	0.3	1.0	0.3	1.0	0.3	1.0
Negative affect	3.7	3.2	3.6	3.1	3.7	3.2
Detachment	6.4	7.0	6.3	6.9	6.5	7.1
Psychoticism	4.0	4.8	4.1	4.9	3.9	4.8
Disinhibition	16.6	6.2	16.5	6.0	16.8	6.3
Externalizing behavior	0.3	0.9	0.3	0.9	0.3	0.9
Savings behavior	4.7	1.3	4.6	1.4	4.7	1.3
Financial distress	1.9	2.8	2.1	2.9	1.8	2.8
Income	7.2	3.6	7.1	3.7	7.3	3.5
Unemployment	0.3	0.7	0.4	0.8	0.3	0.7
Relationship agreement	87.0	10.7	87.6	11.1	86.6	10.4
Legal issues	0.8	1.5	0.8	1.5	0.8	1.5
Occupational citizenship	21.8	8.1	21.7	8.2	21.8	8.0
Counterproductive work behavior	2.8	2.9	2.8	2.9	2.8	2.9
Community attitude	33.9	5.5	33.9	5.5	33.8	5.5
Community behavior	22.7	6.3	22.8	6.4	22.6	6.2
Cognitive ability	8.7	3.8	8.7	3.9	8.6	3.8

*Note*: Phenotypes marked with an asterisk were log-transformed prior to analysis to address skew. Frequency variables were measured with respect to the previous 180 days. Bold indicates significant mean differences between groups at the multiple testing corrected threshold *p* < 0.0017. Rec. refers to recreationally legal states, non-rec. refers to all states without recreational legalization (medical THC, limited medical, decriminalized, and completely illegal states).

The present sample (*N* = 4078) represents 61% of the original samples. Attrition analyses (online Supplementary Table S5) indicated that individuals with higher adolescent externalizing, lower parental education, male sex higher cannabis frequency, higher tobacco frequency, and more nicotine dependence symptoms prior to 2014 were less likely to participate in the joint assessment. There were no significant interactions between predictors of attrition and recruitment site in predicting attrition.

### Population-Level effects of recreational legalization

**Fig. 1.**
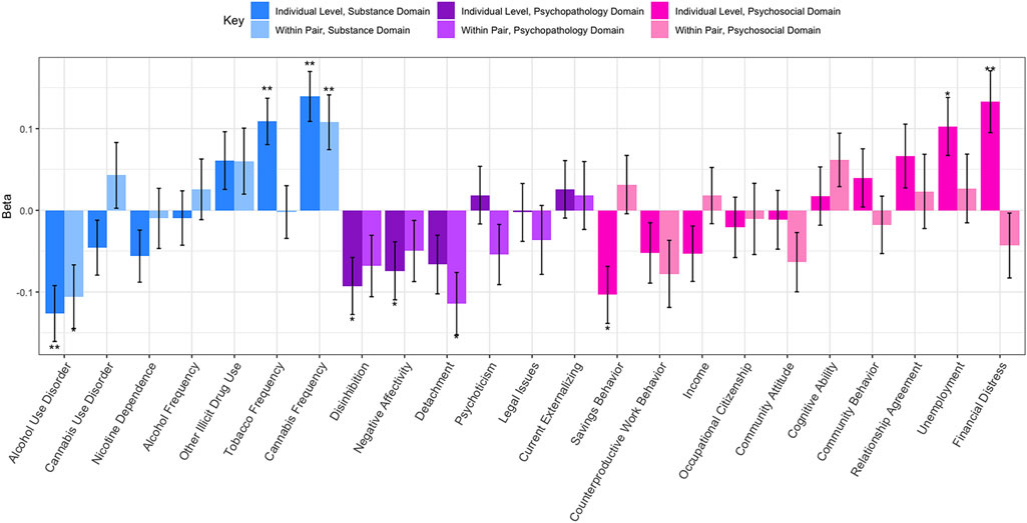
Bar graph depicting the effects sizes of recreational legalization as generated from the individual level and MZ-DZ combined co-twin analyses. One asterisk represents significance at *p* < 0.05 and two asterisks represent significance at the multiple testing corrected threshold *p* < 0.0023. Positive betas indicate increased mean levels in recreational states, negative betas indicate decreased mean levels in recreational states. Notable effects are higher cannabis use frequency within twin pairs for the twin living in a recreationally legal state, as well as decreased AUD symptoms within twin pairs for the individual living in a recreationally legal state. Error bars represent standard error of linear-mixed effects model estimates as generated by the function lmer in the package lme4.

Results from the individual level analyses are presented in Fig. 1. At the individual level, recreational legalization was associated with increased cannabis frequency (*β* = 0.14, s.e. = 0.03, *p* = 5.3 × 10^6^), increased tobacco frequency (*β* = 0.11, s.e. = 0.03, *p* = 1.3 × 10^−4^), decreased alcohol use disorder (AUD) symptoms (*β* = −0.13, s.e. = 0.03, *p* = 2.2 × 10^−4^), and increased financial distress (*β* = 0.13, s.e. = 0.04, *p* = 4.5 × 10^−4^). We were well powered to detect main effects at effect sizes of 0.004 or greater at our adjusted alpha, and there were no other significant effects of legalization.

### Co-twin control analyses

The pooled MZ-DZ co-twin results are presented in Fig. 1 and online Supplementary Table S6 presents the zygosity-stratified results. The effects of legalization on cannabis use frequency and AUD survived the co-twin control analyses with significant within-pair effects, eliminating many potential confounders and alternative explanations for the effects of recreational legalization. The pooled MZ-DZ co-twin control results indicate that cannabis frequency increased by 0.11 standard deviations as a result of recreational legalization (s.e. = 0.03, *p* = 1.3 × 10^−3^). Endorsement of AUD symptoms decreased by 0.11 standard deviations (s.e. = 0.04, *p* = 6.8 × 10^−3^); this value approached but did not surpass our multiple-testing threshold suggesting possibly a spurious result or low power to detect small effects. Comparatively, for financial distress there were no significant within-pair effects (*β*_w_ = −0.04, s.e. = .04, *p* = 0.28), which suggests that the individual level effect may be attributed entirely to unmeasured genetic and environmental confounders. Surprisingly, cannabis use disorder was not associated with legalization, in both the individual-level and within-twin results.

Bootstrap comparisons of *β* and *β*_w_ were computed to evaluate the degree to which the evidence is consistent with a causal explanation, total confounding, or a mixture of causal and confounding influences. Bootstrapped differences revealed minimal attenuation of effect, suggesting evidence consistent with some causal impact of recreational legalization on cannabis use frequency and AUD symptoms (Table 2).

**Table 2.** Results from 1000 bootstrap replicates comparing individual and within-pair effects

Phenotype	Estimates compared	Mean difference	95% Confidence Interval
Cannabis frequency	**Individual – Combined MZ-DZ**	**0.05**	**(0.02–0.09)**
	Individual – MZ Only	0.06	(−0.02 to 0.14)
	Combined MZ-DZ – MZ Only	0.01	(−0.07 to 0.08)
Alcohol use disorder	Individual – Combined MZ-DZ	−0.04	(−0.08 to 0.01)
	Individual – MZ	−0.05	(−0.14 to 0.04)
	Combined MZ-DZ – MZ Only	−0.02	(−0.10 to 0.06)

*Note*: Bootstrap estimates evaluate differences in estimates to evaluate attenuation of effect. The presence of lack of attenuation determines the nature of effect: no attenuation across all estimates indicates evidence consistent with causal influences, some attenuation of effect between individual-combined MZ-DZ and/or combined MZ-DZ – MZ only suggests evidence consistent with mixed causal influences and confounding, total attenuation of effect across all estimates indicates complete confounding. Bold indicates significant mean differences as indexed by 95% confidence intervals that do not overlap 0.

We further investigated the legalization effects on AUD, as we did not hypothesize the identified protective effect of legalization on AUD symptoms, nor did we expect to see differences in AUD without differences in alcohol consumption. As an exploratory follow-up, we examined all 11 alcohol dependence symptoms at the item level, average alcohol quantity when drinking, and maximum drinks in a 24 h period.

Mirroring alcohol frequency, there were no significant effects of recreational legalization on average alcohol quantity at the multiple-testing threshold for either the individual (*β* = −0.10, s.e. = 0.03, *p* = 5.8 × 10^−3^) nor within-pair analyses (*β*_w_ = −0.02, s.e. = 0.04, *p* = 0.67). With respect to maximum drinks in a 24 h period, residents of recreationally legal states drank less than their non-recreational counterparts at the individual level (*β* = −0.14, s.e. = 0.03, *p* = 2.9 × 10^−5^), this effect was strongly attenuated in the co-twin comparison (*β*_w_ = −0.03, s.e. = 0.05, *p* = 0.42) indicating potential genetic and environmental confounding. For AUD symptoms, only one symptom ‘recurrent drinking when physically hazardous’ was significantly associated with recreational legalization in the individual analysis (*β* = −0.62, s.e. = 0.16, *p* = 1.2 × 10^−4^) and this effect persisted in the pooled MZ-DZ co-twin model (*β*_w_ = −0.76, s.e. = 0.19, *p* = 9.0 × 10^−5^). This indicates that those twins living in a recreational-legal state were *less* likely to risk harm while under the influence of alcohol (*N* = 99 endorsements, 6.4% of recreational participants) than their co-twin living in non-recreational states (*N* = 233 endorsements, 9.9% of non-recreational participants). Overall, the results suggest that although the patterns of consumption may not be changed by recreational legalization, physically hazardous drinking is lower in recreationally legal states, even after controlling for unmeasured confounders in the co-twin control design. This pattern was not replicated for cannabis use disorder symptoms at the item level; no CUD item was significantly associated with recreational legalization at the individual level.

### Sensitivity checks

Results are described in detail in the supplement and online Supplementary Tables S7–S11; our results were robust to sensitivity analyses.

### Differential vulnerability

**Fig. 2.**
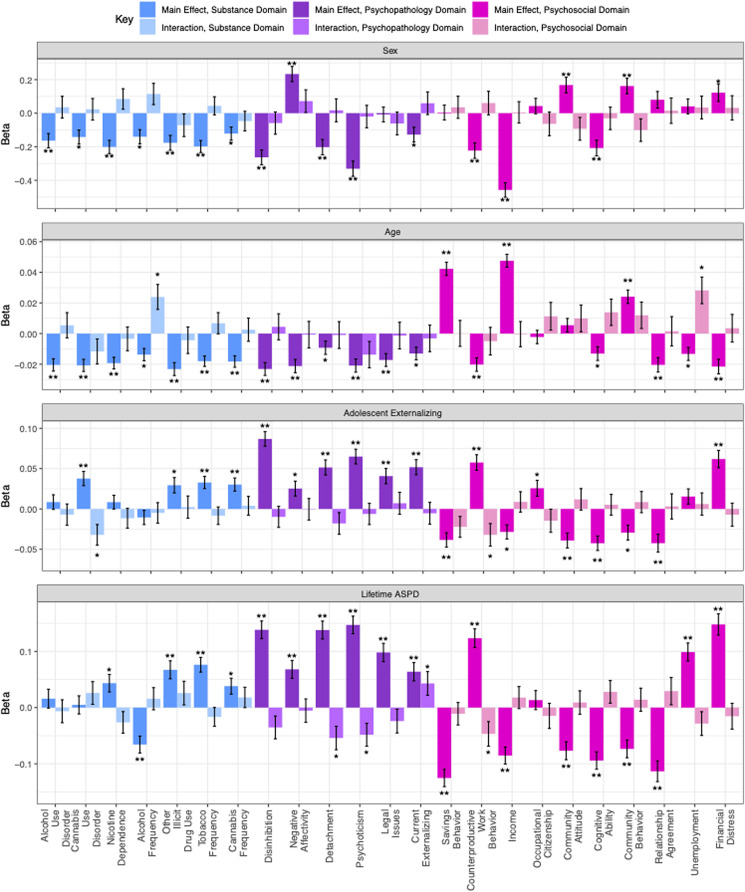
Bar graph depicting the differential vulnerability analyses, indicating the main effects of the risk factors of age, sex (male reference group), adolescent externalizing symptoms, and adult antisocial personality disorder symptoms on the outcomes, as well as the interaction between each risk factor and recreational legalization. One asterisk represents significance at *p* < 0.05 and two asterisks represent significance at the multiple testing corrected threshold of *p* < 0.00046. Positive betas for main effects suggest that the risk factor is associated with increased mean on that outcome (i.e. increased maladaptations); negative betas for main effects indicate the risk factor is associated with decreased mean on that outcome (i.e. decreased adaptations). Positive betas for interaction effects indicate exacerbated risk in legal environments, negative betas for interaction effects indicate mitigated risk in legal environments. Error bars represent standard error of linear-mixed effects model estimates as generated by the function lmer in the package lme4.

To evaluate the existence of differential vulnerability to the effects of recreational legalization, we estimated interaction effects between recreational legalization and indicators of risk: age, sex, adolescent externalizing psychopathology, and adult ASPD. Results are presented in Fig. 2 and online Supplementary Table S12 and posthoc interaction power analyses (Baranger et al., 2022) are presented in online Supplementary Table S13. We were well powered to detect main effects at effect sizes of 0.004 or greater, but we had 80% power to detect interaction effects in only approximately half of the analyses conducted, which was reasonable given the lack of main effects of recreational legalization despite being well powered to detect even small main effects. We were best powered to detect interactions between recreational legalization and adult ASPD or adolescent externalizing (sufficiently powered in 20 and 15 out of 23 analyses respectively) given the strength of correlations between those risk factors and the outcomes. Power to detect interaction effects by sex and age was weaker on average (sufficiently powered in 2 and 7 out of 23 analyses respectively).

For each of the four risk factors, risk generally significantly predicted outcomes in the expected directions (i.e. males at risk for worse externalizing and substance outcomes as compared to females, older individuals at less risk as compared to younger individuals, individuals with higher externalizing symptoms in adolescence or adult ASPD were at high risk as compared to individuals with less symptoms). In contrast, no interaction effect was significant at the adjusted *p* value, thus, we observed no strong evidence of differential vulnerability. Of particular interest are the main effects consistent with causal explanations, i.e., the effect of recreational legalization within pairs on cannabis frequency. We hypothesized men and individuals higher in externalizing would be specifically vulnerable to the effects of legalization; here we found no evidence of interaction by sex (*β* = −0.05, s.e. = 0.06, *p* = 0.42), age (*β* = −0.01, s.e. = 0.01, *p* = 0.74), adolescent externalizing (*β* = 0.004, s.e. = 0.01, *p* = 0.74), or lifetime adult ASPD (*β* = 0.02, s.e. = 0.02, *p* = 0.32) with recreational legalization on cannabis frequency.

## Discussion

We evaluated the effect of recreational legalization on a broad set of outcomes indexing psychosocial dysfunction, as well as how recreational legalization interacts with established risk factors for substance use. We leveraged existing longitudinal twin samples and assessments after legalization to draw potentially causally-informative conclusions about the nature of any legalization effects. Our work replicates and extends existing findings from repeated cross-sectional population studies and controls for additional potential confounds and secular trends.

Using individual and co-twin mixed effects models, we established evidence that suggests cannabis legalization causes a 0.11 standard deviation increase in cannabis frequency, whereas AUD symptoms decreased by 0.11 standard deviations driven by reductions in use of alcohol when physically hazardous. This result for cannabis frequency is consistent with previous repeated cross-sectional research on large population samples (Cerdá et al., 2020; Parnes et al., 2018; Steigerwald et al., 2020; Wallace et al., 2020). The result for AUD is difficult to interpret and merits additional investigation in future work.

Unlike previous work, we did not find increases in cannabis use disorder at the individual level, nor did we detect changes in alcohol or illicit substance consumption (Anderson & Rees, 2021; Cerdá et al., 2020). It is unclear whether the differences between our results and prior work are due to study design, variable definition, sample age, or some other variable. Additionally, though cannabis has been linked to psychotic symptoms, we did not identify a relationship between recreational legalization and psychoticism. Particularly, daily use and/or use of high potency products increases risk for psychotic disorder (Di Forti et al., 2019). It may be that recreational legalization increases use in infrequent or casual users, rather than in heavy users, resulting in no identifiable link between recreational legalization and increased incidence of psychotic symptoms. Future work can more directly examine the relationship between individuals' actual consumption (as opposed to their environment) and these outcomes to continue disentangling the complex causal relationships.

Differential vulnerability analyses indicated that established risk factors for substance use predict psychosocial dysfunction in the expected ways, but these risk factors do not interact with legalization to render some individuals more susceptible to its effects. In other words, individuals at higher risk of substance use problems and other psychosocial dysfunction are not at higher risk in recreationally legal environments as compared to non-recreational environments. This suggests that prevention and intervention efforts may be best implemented by continuing to target established risk factors rather than focusing on availability.

Our hypotheses that men and individuals higher in externalizing would be more strongly impacted by recreational policies were not supported, and unlike previous literature, we did not identify any differential vulnerability by age (Bae & Kerr, 2019; Kerr et al., 2017). This may be due to the different age ranges in each study. The previous works were in undergraduate samples and defined age dichotomously as ‘over or under age 21’. It could be that age effects are most salient for individuals near the legal purchasing age in most recreationally legal states, and that older adults, as in our sample, are not differentially affected by age.

### Limitations

One limitation of our design is the lack of pre-legalization covariates for each outcome variable, which limits our ability to evaluate secular trends in those variables. Furthermore, there are some domains associated with cannabis use that we did not evaluate here, such as physical health, sleep, and motivation, and internalizing diagnoses. Additionally, our sample is an adult community sample broadly characterized by low levels of substance use and psychosocial dysfunction. This limits our ability to generalize relationships between legalization, outcomes, and risk factors for the individuals at greatest risk. Additional research is also needed to understand the effects of legalization on younger individuals when substance use tends to accelerate. Lastly, co-twin models can only account for measured confounds and unmeasured confounds that are shared between twins. While our results suggest cannabis legalization causes changes in cannabis frequency and AUD, there could be unmeasured non-shared confounds that better explain the within-pair effects.

## Conclusions

Recreational cannabis legalization causes increases in mean cannabis frequency and residents of recreational states have fewer recent symptoms of AUD. Broadly speaking, our co-twin control and differential vulnerability results suggest that the impacts of recreational cannabis legalization on psychiatric and psychosocial outcomes are otherwise minimal. We assessed a broad range of outcomes, including other substance use, substance dependence, disordered personality, externalizing and legal issues, relationship agreement, workplace behavior, civic engagement, and cognition and found no detrimental nor protective effects for the majority of these domains, nor did we identify any increased vulnerability conferred by established risk factors.

Both sets of results are reassuring with respect to public health concerns around recreational cannabis legalization. This does not, however, imply that cannabis consumption is without risk, only that we do not identify meaningful changes in these negative outcomes as a result of legalization. Future research can expand to additional outcomes, alternative risk factors or moderators, and more diverse demographic groups to ensure a broad understanding of the consequences and benefits of cannabis legalization. Additionally, cannabis legalization has resulted in a wider range of easily available products with varying routes of administration (edibles, tinctures, concentrates) and products are increasing in potency over time (Chandra et al., 2019). High THC potency is associated with increased risk of mental health problems, as compared to lower THC potency (Hines et al., 2020; Petrilli et al., 2022). Future work incorporating standardized dosages (Volkow & Weiss, 2020) and varied routes of administration may provide us with additional nuance in how cannabis consumption relates to adverse outcomes and what patterns of consumption are associated with the smallest and largest risks.

## Supplementary material

For supplementary material accompanying this paper visit https://doi.org/10.1017/S0033291722003762. click here to view supplementary material
