# Supplementary material for: Recreational cannabis legalization has had limited effects on a wide range of adult psychiatric and psychosocial outcomes
Source: Psychol Med. 2023 Jan 5;53(14):6481–90. doi: 10.1017/S0033291722003762 (PMC10319916; doi:10.1017/S0033291722003762)
Supplement: Supplementary file 1 [file S0033291722003762sup001.docx]

Supplemental Materials: Recreational Cannabis Legalization has had Limited Effects on a Wide Range of Adult Psychiatric and Psychosocial Outcomes 

Stephanie M. Zellers^1, 2*^

J. Megan Ross^3^

Gretchen R. B. Saunders^1^

Jarrod M. Ellingson^3, 4^

Tasha Walvig^1^

Jacob E. Anderson^1^

Robin P. Corley^4^

William Iacono^1^

John K. Hewitt^4, 5^

Christian J. Hopfer^3, 4^

Matt K. McGue^1^

 Scott Vrieze^1^

1. Department of Psychology, University of Minnesota, Minneapolis, Minnesota, USA

2. Institute for Molecular Medicine Finland, University of Helsinki, Helsinki, Finland

3. Department of Psychiatry, University of Colorado Anschutz Medical Campus, Aurora, Colorado, USA

4. Institute for Behavioral Genetics, University of Colorado Boulder, Boulder, Colorado, USA

5. Department of Psychology and Neuroscience, University of Colorado Boulder, Boulder, Colorado, USA

Correspondence concerning this article should be addressed to Stephanie Zellers, Institute for Molecular Medicine Finland, University of Helsinki, P.O. Box 20 Helsinki, Finland 00014 Contact: stephanie.zellers@helsinki.fi

**Supplementary Methods**

***Pre-2014 Harmonization***

*Rearing SES/Parental Education*

Parental education was measured via structured clinical interview. Both Colorado and Minnesota participants’ biological parents reported on their education at intake using the Hollingshead Four Factor Index of Socioeconomic Status. In Colorado, education level was reported as 1 = grade school, 2 = some high school, 3 = high school or GED, 4 = technical school, 5 = some college, 6 = college graduate, 7 = graduate or professional degree. In Minnesota, education level was reported as 1 = less than high school, 2 = high school, 3 = GED, 4 = technical degree, 5 = business certificate, 6 = associates or some college, 7 = BA/BS, 8 = graduate or professional degree. Table S1 presents mappings to harmonize these categories. Variables were first recoded to match scales, then biological maternal and paternal reports were averaged to create a parental education measure. If one biological parent was missing a report, we used the valid parental data as the parental education measure. 

*Cannabis Frequency*

Cannabis frequency was measured via structured clinical interview. Cannabis use was operationalized in two ways, to reflect earlier Minnesota and Colorado assessments. We used both operationalizations to evaluate robustness of findings across the two cannabis use measures described below. For earlier Colorado assessments, cannabis use frequency was assessed in an interview as a continuous number of days used in last 180. For earlier MN assessments, cannabis use frequency was assessed in an interview as average frequency of use in the last 12 months. Response options included less than once a year, less than once a month but at least once a year, about once a month, 2 or 3 times a month, 1 or 2 times a week, 3 to 4 times a week, nearly every day, every day, 2 times a day, and 3 or more times a day. Responses were binned to collapse daily use categories and collapse non-users with individuals using less than once a year (i.e., not within the last 12-months). Tables S2 and S3 have the mappings from earlier Colorado to Minnesota responses and vice versa. 

*Alcohol Use Frequency*

Alcohol frequency was measured via structured clinical interview. Alcohol use was operationalized in two ways, to reflect earlier Minnesota and earlier Colorado assessments. We used both operationalizations to evaluate robustness of findings across the two alcohol use measures described below. For earlier Colorado assessments, alcohol use frequency was assessed in an interview as a continuous number of days used in last 180. For earlier Minnesota assessments, alcohol use frequency was assessed in an interview as average frequency of use in the last 12 months. Response options included less than once a year, less than once a month but at least once a year, about once a month, 2 or 3 times a month, 1 or 2 times a week, 3 to 4 times a week, nearly every day, every day, 2 times a day, and 3 or more times a day. Responses were binned to collapse daily use categories and collapse non-users with individuals using less than once a year (i.e., not within the 12-month reporting window). To harmonize earlier Colorado responses to Minnesota bins, and Minnesota responses to Colorado values, we used the same mappings as presented in Tables 2 and 3 for cannabis frequency, as the item wordings are the same. 

*Tobacco Frequency*

Tobacco frequency was measured via structured clinical interview. For earlier Colorado assessments, tobacco use frequency was also assessed as a continuous number of days used in last 180 (response options ranging from 0-180) in an interview. This is the same as the joint assessment item, so no harmonization is needed. For earlier Minnesota assessments, tobacco use frequency was assessed in an interview as days per typical month in which they used tobacco products in the last 12 months (response options ranging from 0-30). To harmonize earlier Minnesota responses to Colorado and the joint assessment, we multiplied the Minnesota “number of days used in a typical month” value by 6 to reach an approximation for use in the last 180 days.  

*Adolescent Externalizing*

Adolescent externalizing psychopathology was operationalized as harmonized symptom counts of ADHD and conduct disorder across DSM-III-R and IV from a structured clinical interview. We have previously used these symptom counts in related prior work and the symptom inclusion/exclusions are listed in the supplement of the published article Zellers et al. (2020). We utilized the earliest assessment available for ADHD symptoms and earliest assessment available between ages 15 and 21 for conduct disorder; these ages were chosen based on the ages of onset and symptoms for each disorder. This measure was designed to investigate premorbid risk, measured at ages before the majority of the sample initiates substance use. 

*Lifetime Clinical Measures: Substance Abuse and Dependence, Antisocial Personality Disorder*

At earlier assessments, Minnesota used a structured clinical interview with DSM-III-R criteria to assess lifetime symptoms at intake and then at each later assessment, as well as symptoms present since last assessment (i.e., in the last 3 years, last 6 years, etc.). At earlier assessments, Colorado assessed lifetime symptoms using a structured clinical interview with DSM-IV criteria. Given differences in DSM version and reporting window, earlier clinical measures were harmonized for use as a covariate. 

To harmonize earlier measurements, we first reviewed the symptoms in each version of the DSM for equivalence and include only the subset of symptoms deemed to be equivalent across DSM-III-R and DSM-IV. Then to address differences in reporting windows, we created a “lifetime total” across assessments for each measure by evaluating whether each included symptom was ever endorsed by a participant at any pre-2014 assessment and summing the total number of pre-2014 “ever-endorsed” symptoms. We created a lifetime pre-2014 “ever diagnosis” based on whether the individual met diagnostic criteria at any prior assessment. These measures were intended to evaluate risk lifetime, or after the initiation of substance use.

For alcohol, nicotine, and cannabis dependence, included symptoms are as follows: taken a larger amount or for longer than intended, unsuccessful desire to control use, much time spent obtaining substances or recovering from substance use, forgoing important activities, continued use despite problems, and withdrawal. Alcohol and cannabis symptom totals also included failure in work/school/home obligations, use when physically hazardous, and tolerance; these three were not uniformly assessed between sites for nicotine dependence, as two of these three represent symptoms of abuse. This resulted in a maximum of 6 symptoms of nicotine dependence and 9 symptoms of alcohol or cannabis abuse/dependence. 

For antisocial personality disorder, the 7 symptoms approximately equivalent between versions are as follows: failure to conform to social norms with respect to lawful behavior, deceitfulness and lack of regard for the truth, impulsivity, irritability and aggression, reckless disregard for the safety of others, consistent irresponsibility with regards to work or financial obligations, and lack of remorse. 

**Post-2014 Psychosocial Outcomes**

*Cannabis Frequency*

Cannabis frequency was measured via online self-report survey. Cannabis frequency items are described in the pre-2014 measures section. Both the ordinal and continuous cannabis use frequency items were presented to all jointly assessed participants as a part of the self-report survey. 

*Alcohol Use Frequency*

Alcohol frequency was measured via online self-report survey. Alcohol frequency items are described in the pre-2014 measures section. Both the ordinal and continuous alcohol use frequency items were presented in all self-report survey joint assessments.  

*Alcohol Use Quantity*

	Alcohol quantity was reported continuously as a free response where participants could indicate their average drinks per drinking occasion in the last 12 months (one beer, one glass of wine, one shot of liquor, or one mixed drink). Any responses greater than 30 drinks were recoded as 30.

*Maximum Drinks in 24 Hours*

	Maximum drinks were reported continuously as a free-response where participants could indicate the most drinks they had in any one 24-hour period during the past 12 months. Any responses greater than 30 drinks were recoded as 30. 

*Tobacco Frequency*

Tobacco frequency was measured via online self-report survey. Tobacco use will be operationalized as a continuous “number of days used in the last 180”. This is the primary tobacco frequency item available in the post-2014 self-report survey joint assessments.

*Other Drug Use*

Other drug use was measured via online self-report survey. Other drug use in the last year was operationalized as a continuous “number of non-prescribed other drugs besides alcohol, tobacco, and cannabis” that the participants report using in the last 12 months. “Other drugs” includes stimulants, barbiturates/sedatives/tranquilizers, cocaine, heroin, other opiates, synthetic marijuana/spice, PCP, psychedelics/hallucinogens, MDMA, Ketamine, inhalants/gas, and steroids. 

*Last 12-Months Clinical Measures: Substance Dependence and Antisocial Personality Disorder*

In post-2014 joint assessments, the clinical measures were assessed in the same way for all participants using DSM-5. Current problematic substance use (alcohol, tobacco, and cannabis) and antisocial personality disorder were operationalized as past 12-month symptom counts and last 12 months diagnosis generated from a structured clinical interview. 

*Personality* 

Disordered personality traits were measured via online self-report survey. Disordered personality traits were assessed using abbreviated (4-item) self-report scales from the PID-5 (American Psychiatric Association et al., 2015). The following scales were assessed: anxiousness, anhedonia, depressivity, intimacy avoidance, withdrawal, eccentricity, perceptual dysregulation, unusual beliefs and experiences, distractibility, impulsivity, irresponsibility, lack of rigid perfectionism, and risk taking. Scores for the 13 PID-5 scales were taken by summing the scores of the 4 items scores, after any necessary reverse coding. Individuals were allowed to miss no more than 1 item per scale, with proration of the missing item based on mean of other 3 items. These scales correspond to four factors: negative affectivity, detachment, psychoticism, and disinhibition. Negative affectivity is comprised of the anxiousness scale. Detachment is comprised of the withdrawal, anhedonia, depressivity, and intimacy avoidance scales. Psychoticism is comprised of the unusual beliefs and experiences, perceptual dysregulation, and eccentricity scales. Disinhibition is comprised of the irresponsibility, impulsivity, lack of rigid perfectionism, distractibility, and risk taking scales, 

*Externalizing Behavior*

Externalizing behavior was measured via online self-report survey. Externalizing behavior was assessed using a self-report of the Brief Form of the Externalizing Behavior Inventory (Patrick, Kramer, Krueger, & Markon, 2013). Externalizing Behavior Inventory scores allowed one missing item per scale except for the 29-item total score, where 3 missing items (i.e., 10%) were allowed. Scales include theft, fraud, physical aggression, destructive aggression, and a total score was created as well. 

*Finances*

Finances were measured via online self-report survey. Finances were assessed as two scales: saving disposition and financial distress/problems. Scales were comprised of yes/no items; saving disposition items were assessed for their current situation or beliefs (ex. do you regularly save some of the money you earn by placing it in a special account), financial distress (ex. have you been homeless) and problems (ex. have you found it difficult to meet the cost of things like having a night out or presents for the family) were assessed with respect to last 12 months. 

Income was also measured via online self-report survey. Income was assessed as annual gross income before taxes from the participants’ job on a 1-16 ordinal scale where 1 reflects no paying job and 16 reflects 200,000 dollars or more. Steps between response options were 9,999 dollars from 0-99,999 dollars to 24,999 dollars from 100,000 to 200,000 or more dollars.  

*Employment*

Employment was measured via online self-report survey. An employment scale was derived as the sum of 5 items to determine degree of unemployment. Individuals reported whether they were 1) employed or self-employed at any time in the last 12 months, 2) currently employed or self-employed, 3) had been unemployed for at least 3 months in the last 12, 4) laid off or fired in the last 12 months, or 5) demoted at work in the last 12 months. Higher scores indicate greater degrees of unemployment.

*Relationships*

Relationship agreement was measured via online self-report survey. Relationships are assessed in participants who were married, living together, or in a committed romantic relationship. Relationships were assessed with a scale based on the Dyadic Adjustment Scale (Spanier & Thompson, 1982) with additional items (18 items) where higher scores indicate more agreement in relationships (ex. how often do my partner and I agree on amount of time spent together). Participants responded on a 0-5 scale indicating how frequently each event or disagreement occurred in the last 12 months, with a total score created after reverse coding some items. We also assessed a four item “drugs disagreement” scale where higher scores correspond to more disagreement about drug use (ex. how often do my partner and I disagree about my use of alcohol). The items were scored as a drug-related agreement total, non-drug related agreement total, and an overall agreement score. 

*Legal Matters*

Legal involvement was measured via online self-report survey. Legal matters are assessed as drug (12 items, ex. I received a citation for driving under the influence of alcohol) and non-drug (14 items, ex. I had to go to court) related matters, as well as a total score. Participants responded on an ordinal scale where 0=not at all, 1=once, 2=2-3 times, 3=4 or more times.

*Citizenship and Workplace Behaviors*

Workplace behaviors were measured via online self-report survey. Workplace behaviors were assessed in participants with full or part time jobs with short versions of the Occupational Citizenship Behavior Checklist and the Counterproductive Work Behavior Checklist, both with ten items (Spector, Bauer, & Fox, 2010). Higher total scores on the Occupational Citizenship Behavior Checklist reflect greater prosocial work behaviors (ex. I helped a coworker who had too much to do), whereas high scores on the Counterproductive Work Behavior Checklist reflect antisocial or problematic work behaviors (ex. I came to work late without permission). 

*Civic Engagement*

Civic engagement was measured via online self-report survey. Civic engagement was assessed as attitudes and behaviors within the participants’ communities with items adapted from the Civic Engagement Scale (Doolittle & Faul, 2013). Higher scores on the attitudes scale indicate a sense of shared responsibility to the community (ex. I believe it is important to volunteer), whereas higher scores on the behaviors scale indicate more measurable actions related to community and political engagement (ex. I stay informed of events in my community). 

*Cognition*

Cognitive ability was measured via online self-report survey. General cognitive ability was assessed with the International Cognitive Ability Resource (Condon & Revelle, 2014). This measure has four 4-item facets: verbal reasoning, letter/number series, matrix reasoning, and rotations in three dimensions. There is also a total score across all four facets. Missing items were treated as incorrect unless the participants missed more than half the items on a scale, in which case scales were coded as missing. Higher scores indicate greater general cognitive ability. 

***Sensitivity Analyses***

	To further evaluate the effect of attrition, we ran sensitivity analyses utilizing inverse propensity score weighting. We first fitted multiple predictive models, with varying combinations of the individual variables predicting attrition, to select a single model for inverse propensity score generation. The best predictive model was selected using the R package “performance” (Lüdecke, Ben-Shachar, Patil, Waggoner, & Makowski, 2021). We selected the best model on the basis of effect sizes, AIC and BIC, amount of missing data on the predictors, and variance explained by the fixed effects and full model. This best model was then used to generate the inverse propensity score weights, which were then scaled within twin-pairs. The resulting scaled inverse propensity scores were incorporated into the individual level mixed effects models using the weights function in the lmer command.

	As a sensitivity check on the variable harmonizations, we evaluated ordinal measures of substance consumption and binary diagnoses of substance dependence. We also completed a sensitivity check for our definition of “recreationally legal”. As recreational policies are enacted at the state level, there are substantial variations in policies and enactment dates. We therefore ran an analysis with a stricter definition of recreational legalization, in which a state must have had a recreationally legal policy and open recreational dispensaries for the entirety of our data collection period in order to be classified as recreationally legal. This exposure can be described as “exposure to recreational dispensaries”.

	We also conducted sensitivity checks in which models were run without longitudinal covariates and compared to the primary models that contained covariates. Additionally, as there was considerable range in pre-legalization test year for those pre-legalization covariates sourced from only one assessment (cannabis, alcohol, and tobacco frequency), we conducted a sensitivity analysis covarying for pre-legalization assessment year for those three outcomes.  Lastly, we completed exploratory analyses on income and employment outcomes by including a covariate for global exposure to Covid-19 (i.e., was the assessment prior to or on/after March 13, 2020, when the United States federal government declared Covid-19 to be a public health emergency). 

**Supplementary Results**

To further investigate the effect of attrition we ran weighted models and compared results to the unweighted models in the main text. Results are presented in Table S7; none of the substantive conclusions changed with respect to the significance and direction of effect identified in the primary analysis. 

To evaluate sensitivity of results to our definition of recreational policy change dates, we reran all individual and co-twin models with a stricter definition of recreationally legal: open recreational dispensaries for the entire duration of data collection. Results are presented in Table S8; none of the substantive conclusions changed with respect to the significance and direction of effect identified in the primary analysis. 

	Results of the sensitivity analyses on ordinal measures of consumption, binary diagnoses, and analyses altering the longitudinal covariates are presented in Tables S9 and S10. All effects reported in the main text were robust to these changes. Lastly, supplemental analyses including a covariate for nationwide exposure to Covid-19 are presented in Table S11. Covid-19 did significantly predict negative financial events, income, and employment, but no changes were observed to the significance and direction of recreational legalization effects as compared to the primary analyses. 

References

American Psychiatric Association, CDC, Krueger, R. F., Derringer, J., Markon, K. E., Watson, D., … Sartorius, N. (2015). PID-5 English. *World Psychiatry : Official Journal of the World Psychiatric Association (WPA)*. doi: 10.1002/wps.20231

Condon, D. M., & Revelle, W. (2014). The international cognitive ability resource: Development and initial validation of a public-domain measure. *Intelligence*, *43*, 52–64. doi: 10.1016/j.intell.2014.01.004

Doolittle, A., & Faul, A. C. (2013). Civic Engagement Scale: A Validation Study. *SAGE Open*, *3*(3), 1–7. doi: 10.1177/2158244013495542

Lüdecke, D., Ben-Shachar, M. S., Patil, I., Waggoner, P., & Makowski, D. (2021). performance: An R Package for Assessment, Comparison and Testing of Statistical Models. *Journal of Open Source Software*, *6*(60), 3139. doi: 10.21105/joss.03139

Patrick, C. J., Kramer, M. D., Krueger, R. F., & Markon, K. E. (2013). Optimizing Efficiency of Psychopathology Assessment through Quantitative Modeling: Development of a Brief Form of the Externalizing Spectrum Inventory. *Psychological Assessment*, *25*(4), 1332. doi: 10.1037/A0034864

Spanier, G. B., & Thompson, L. (1982). A Confirmatory Analysis of the Dyadic Adjustment Scale. *Journal of Marriage and the Family*, *44*(3), 731. doi: 10.2307/351593

Spector, P. E., Bauer, J. A., & Fox, S. (2010). Measurement artifacts in the assessment of counterproductive work behavior and organizational citizenship behavior: Do we know what we think we know? *Journal of Applied Psychology*, *95*(4), 781–790. doi: 10.1037/A0019477

| Table S1: Recoding Colorado and Minnesota Parental Education Scales to Harmonize Responses |  |  |
| --- | --- | --- |
| Original Colorado Value | **Original Minnesota Value** | **Harmonized Response** |
| Grade school | Less than high school | Less than high school |
| Some high school |  |  |
| High School or GED | High school | High school or GED |
|  | GED |  |
| Technical school | Technical degree | Technical degree |
| Some college | Associates or some college | Associates or some college or Business certificate |
|  | Business certificate |  |
| College Graduate | BA/BS | BA/BS college graduate |
| Graduate or Professional Degree | Graduate or professional degree | Graduate or professional degree |

| Table S2: Recoding Substance Frequency Responses |  |
| --- | --- |
| Colorado Original Continuous Response | **Recoded Response to Match Minnesota** |
| No lifetime use | Did not use in the last year |
| Lifetime use but no use in reporting window | Did not use in the last year |
| 1-3 days | Less than once a month, at least once a year |
| 4-6 days | About once a month |
| 7-19 days | 2 or 3 times a month |
| 20-54 days | 1 or 2 times a week |
| 55-99 days | 3 or 4 times a week |
| 100-179 days | Nearly every day |
| 180 days | Daily |
| Minnesota Original Ordinal Response | **Recoded response to match Colorado** |
| Did not use in the last year | 0 |
| Less than once a month, at least once a year | 3 |
| About once a month | 6 |
| 2 or 3 times a month | 15 |
| 1 or 2 times a week | 39 |
| 3 or 4 times per week | 91 |
| Nearly every day | 150 |
| Daily | 180 |

| Table S3: Ordinal Frequency Means and Diagnostic Endorsement Rates for Sensitivity Analyses |  |  |  |
| --- | --- | --- | --- |
| Phenotype | **Full Sample Mean (SD)** | **Legal Mean (SD)** | **Illegal Mean (SD)** |
| Ordinal Cannabis Frequency | 1.0 (2.0) | 1.2 (2.2) | 0.9 (1.9) |
| Ordinal Alcohol Frequency | 3.1 (2.0) | 3.1 (2.0) | 3.2 (1.9) |
| Phenotype | **Full Sample Endorsement (%)** | **Legal Endorsement (%)** | **Illegal Endorsement** **(%)** |
| Cannabis Use Disorder | 313 (7.9%) | 131 (8.4%) | 180 (7.6%) |
| Alcohol Use Disorder | 788 (19.9%) | 277 (17.7%) | 500 (21.1%) |
| Nicotine Dependence | 555 (14.0%) | 197 (12.6%) | 351 (14.8%) |

Note: Legal and illegal endorsements do not sum to full sample endorsement

due to missingness on residence item

| Table S4: Twin Correlations by Residence |  |  |  |  |  |  |
| --- | --- | --- | --- | --- | --- | --- |
|  | **MZ Concordant Legal** | **DZ Concordant Legal** | **MZ Concordant Illegal** | **DZ Concordant Illegal** | **MZ Discordant** | **DZ Discordant** |
| Alcohol Use Disorder Symptoms | 0.38 [0.26, 0.49],   N = 227 | 0.05 [-0.12, 0.22],   N = 136 | 0.28 [0.19, 0.36],   N = 477 | 0.1 [-0.03, 0.22],   N = 254 | 0.19 [-0.02, 0.39],   N = 87 | -0.06 [-0.33, 0.22],   N = 52 |
| Cannabis Use Disorder Symptoms | 0.07 [-0.06, 0.2],   N = 224 | 0.24 [0.07, 0.39],   N = 134 | 0.36 [0.28, 0.44],   N = 472 | 0.3 [0.18, 0.4],   N = 255 | -0.04 [-0.25, 0.17],   N = 87 | -0.04 [-0.32, 0.23],   N = 51 |
| Nicotine Use Disorder Symptoms | 0.23 [0.11, 0.35],   N = 228 | -0.02 [-0.19, 0.15],   N = 136 | 0.4 [0.33, 0.48],   N = 477 | 0.05 [-0.08, 0.17],   N = 255 | 0.13 [-0.08, 0.33],   N = 88 | -0.01 [-0.28, 0.26],   N = 52 |
| Current Nicotine Frequency | 0.39 [0.28, 0.49],   N = 248 | 0.19 [0.03, 0.35],   N = 141 | 0.55 [0.48, 0.61],   N = 485 | 0.15 [0.03, 0.27],   N = 261 | 0.5 [0.33, 0.64],   N = 93 | 0.18 [-0.09, 0.44],   N = 52 |
| Current Alcohol Frequency | 0.43 [0.32, 0.53],   N = 249 | 0.19 [0.02, 0.34],   N = 141 | 0.47 [0.4, 0.54],   N = 484 | 0.24 [0.12, 0.35],   N = 262 | 0.41 [0.22, 0.57],   N = 93 | 0.31 [0.04, 0.54],   N = 52 |
| Current Cannabis Frequency | 0.46 [0.35, 0.55],   N = 249 | 0.24 [0.08, 0.39],   N = 141 | 0.44 [0.36, 0.51],   N = 485 | 0.27 [0.16, 0.38],   N = 262 | 0.17 [-0.04, 0.36],   N = 93 | 0.31 [0.04, 0.53],   N = 52 |
| Illicit Drug Use | 0.25 [0.13, 0.36],   N = 247 | 0.24 [0.07, 0.39],   N = 141 | 0.33 [0.25, 0.41],   N = 482 | 0.04 [-0.08, 0.16],   N = 262 | 0.35 [0.16, 0.52],   N = 93 | 0.7 [0.52, 0.82],   N = 52 |
| Current Externalizing Behavior | 0.2 [0.08, 0.32],   N = 247 | -0.03 [-0.2, 0.13],   N = 141 | 0.23 [0.14, 0.31],   N = 483 | 0.23 [0.11, 0.34],   N = 262 | 0.13 [-0.08, 0.32],   N = 93 | -0.04 [-0.31, 0.23],   N = 52 |
| Legal Issues | 0.21 [0.08, 0.32],   N = 248 | 0.04 [-0.13, 0.2],   N = 141 | 0.18 [0.09, 0.26],   N = 483 | 0.11 [-0.01, 0.23],   N = 262 | 0.26 [0.06, 0.44],   N = 93 | 0.16 [-0.11, 0.42],   N = 52 |
| Occupational Citizenship | 0.18 [0.05, 0.32],   N = 200 | -0.03 [-0.21, 0.14],   N = 122 | 0.25 [0.16, 0.34],   N = 434 | 0.15 [0.02, 0.28],   N = 224 | 0.14 [-0.09, 0.36],   N = 75 | 0.25 [-0.04, 0.5],   N = 48 |
| Counter-productive Work Behavior | 0.3 [0.17, 0.43],   N = 199 | 0.12 [-0.06, 0.29],   N = 122 | 0.34 [0.25, 0.42],   N = 434 | 0.19 [0.06, 0.31],   N = 224 | 0.22 [0, 0.43],   N = 75 | 0.29 [0.01, 0.53],   N = 48 |
| Unemployment Index | 0.19 [0.06, 0.3],   N = 249 | 0.17 [0.01, 0.33],   N = 141 | 0.25 [0.16, 0.33],   N = 486 | 0.03 [-0.09, 0.15],   N = 262 | 0.03 [-0.17, 0.24],   N = 94 | 0.26 [-0.01, 0.5],   N = 52 |
| Community Engagement Attitudes | 0.44 [0.34, 0.54],   N = 247 | 0.24 [0.08, 0.39],   N = 141 | 0.48 [0.41, 0.54],   N = 482 | 0.31 [0.19, 0.41],   N = 262 | 0.2 [0, 0.39],   N = 93 | 0.27 [0, 0.51],   N = 52 |
| Community Engagement Behaviors | 0.5 [0.4, 0.58],   N = 246 | 0.3 [0.15, 0.45],   N = 141 | 0.53 [0.46, 0.59],   N = 482 | 0.25 [0.13, 0.36],   N = 262 | 0.36 [0.17, 0.52],   N = 93 | 0.33 [0.06, 0.55],   N = 52 |
| ICAR | 0.62 [0.54, 0.69],   N = 247 | 0.31 [0.16, 0.46],   N = 139 | 0.6 [0.53, 0.65],   N = 482 | 0.24 [0.13, 0.36],   N = 262 | 0.65 [0.51, 0.75],   N = 93 | 0.2 [-0.08, 0.45],   N = 51 |
| Savings | 0.48 [0.38, 0.57],   N = 248 | 0.35 [0.2, 0.49],   N = 141 | 0.45 [0.38, 0.52],   N = 479 | 0.28 [0.16, 0.39],   N = 261 | 0.41 [0.22, 0.57],   N = 93 | 0.24 [-0.04, 0.48],   N = 52 |
| Income | 0.53 [0.43, 0.61],   N = 245 | 0.29 [0.13, 0.44],   N = 141 | 0.55 [0.48, 0.61],   N = 471 | 0.37 [0.26, 0.47],   N = 257 | 0.48 [0.3, 0.62],   N = 93 | 0 [-0.27, 0.28],   N = 52 |
| Relationship Agreement | 0.25 [0.1, 0.39],   N = 159 | 0.13 [-0.08, 0.32],   N = 90 | 0.18 [0.07, 0.28],   N = 325 | 0.19 [0.04, 0.33],   N = 166 | 0.09 [-0.16, 0.32],   N = 68 | -0.1 [-0.41, 0.23],   N = 38 |
| Financial Negative Events | 0.38 [0.27, 0.48],   N = 245 | 0.19 [0.02, 0.34],   N = 141 | 0.44 [0.34, 0.52],   N = 311 | 0.28 [0.13, 0.41],   N = 179 | 0.35 [0.15, 0.52],   N = 88 | 0.34 [0.06, 0.57],   N = 48 |
| Negative Affect | 0.47 [0.36, 0.56],   N = 246 | 0.01 [-0.15, 0.18],   N = 141 | 0.41 [0.33, 0.48],   N = 479 | 0.22 [0.11, 0.34],   N = 261 | 0.34 [0.14, 0.51],   N = 93 | 0.35 [0.09, 0.57],   N = 52 |
| Detachment | 0.37 [0.25, 0.47],   N = 246 | 0.1 [-0.07, 0.26],   N = 141 | 0.4 [0.33, 0.48],   N = 479 | 0.17 [0.05, 0.28],   N = 261 | 0.48 [0.3, 0.62],   N = 93 | 0.04 [-0.24, 0.32],   N = 51 |
| Psychoticism | 0.46 [0.35, 0.55],   N = 246 | 0.12 [-0.04, 0.28],   N = 141 | 0.47 [0.39, 0.53],   N = 478 | 0.24 [0.13, 0.35],   N = 261 | 0.54 [0.37, 0.67],   N = 93 | 0.12 [-0.16, 0.38],   N = 51 |
| Disinhibition | 0.44 [0.34, 0.54],   N = 246 | 0.16 [0, 0.32],   N = 141 | 0.5 [0.43, 0.56],   N = 479 | 0.23 [0.11, 0.34],   N = 261 | 0.41 [0.23, 0.57],   N = 93 | 0.27 [0, 0.51],   N = 51 |
| Pre-2014 Lifetime Cannabis Use Disorder Symptoms | 0.43 [0.32, 0.52],   N = 249 | 0.22 [0.05, 0.37],   N = 141 | 0.59 [0.53, 0.64],   N = 486 | 0.35 [0.24, 0.45],   N = 262 | 0.29 [0.09, 0.46],   N = 94 | 0.58 [0.37, 0.74],   N = 52 |
| Pre-2014 Lifetime Alcohol Use Disorder Symptoms | 0.62 [0.54, 0.69],   N = 249 | 0.25 [0.09, 0.4],   N = 141 | 0.56 [0.49, 0.61],   N = 486 | 0.49 [0.4, 0.58],   N = 262 | 0.67 [0.54, 0.77],   N = 94 | 0.4 [0.14, 0.6],   N = 52 |
| Pre-2014 Lifetime Nicotine Use Disorder Symptoms | 0.68 [0.61, 0.74],   N = 249 | 0.36 [0.2, 0.49],   N = 141 | 0.64 [0.58, 0.69],   N = 486 | 0.4 [0.29, 0.5],   N = 262 | 0.72 [0.6, 0.8],   N = 94 | 0.31 [0.04, 0.54],   N = 52 |
| Pre-2014 Cannabis Frequency | 0.4 [0.29, 0.5],   N = 247 | 0.47 [0.33, 0.59],   N = 139 | 0.54 [0.47, 0.6],   N = 454 | 0.29 [0.17, 0.41],   N = 240 | 0.28 [0.08, 0.46],   N = 92 | 0.72 [0.55, 0.83],   N = 50 |
| Pre-2014 Alcohol Frequency | 0.4 [0.29, 0.5],   N = 247 | 0.32 [0.16, 0.46],   N = 139 | 0.5 [0.43, 0.56],   N = 476 | 0.3 [0.18, 0.4],   N = 260 | 0.46 [0.29, 0.61],   N = 93 | 0.54 [0.31, 0.71],   N = 52 |
| Pre-2014 Tobacco Frequency | 0.43 [0.32, 0.53],   N = 247 | 0.17 [0, 0.33],   N = 139 | 0.62 [0.56, 0.67],   N = 472 | 0.38 [0.27, 0.48],   N = 259 | 0.42 [0.24, 0.58],   N = 93 | 0.35 [0.09, 0.57],   N = 52 |
| Adolescent Externalizing Symptoms | 0.51 [0.41, 0.6],   N = 236 | 0.13 [-0.04, 0.29],   N = 134 | 0.43 [0.36, 0.5],   N = 480 | 0.36 [0.25, 0.46],   N = 254 | 0.37 [0.17, 0.54],   N = 89 | 0.53 [0.3, 0.71],   N = 50 |
| Pre-2014 Lifetime Antisocial Symptoms | 0.48 [0.37, 0.57],   N = 249 | 0.28 [0.12, 0.43],   N = 141 | 0.67 [0.62, 0.72],   N = 486 | 0.53 [0.44, 0.61],   N = 262 | 0.6 [0.45, 0.72],   N = 94 | 0.55 [0.32, 0.71],   N = 52 |

Note: Correlations are Pearson Product-Moment Correlations computed on pairwise complete observations.  

| Table S5: Factors Predicting Attrition |  |  |  |  |  |  |
| --- | --- | --- | --- | --- | --- | --- |
|  | **Main Effect on Attrition** |  |  | **Interaction with Site** |  |  |
|  | **Beta** | **SE** | **P** | **Beta** | **SE** | **P** |
| Pre-2014 Last 12 Months Marijuana Frequency | **-0.11** | **0.03** | **1.1E-03** | 0.22 | 0.08 | 5.9E-03 |
| Pre-2014 Last 12 Months Alcohol Frequency | -0.01 | 0.03 | 0.67 | -0.04 | 0.08 | 0.62 |
| Pre-2014 Last 12 Months Tobacco Frequency | **-0.18** | **0.02** | **4.4E-17** | 0.15 | 0.12 | 0.19 |
| Pre-2014 Lifetime CUD Symptoms | -0.06 | 0.03 | 0.02 | -0.03 | 0.09 | 0.71 |
| Pre-2014 Lifetime AUD Symptoms | -0.01 | 0.02 | 0.56 | -0.25 | 0.11 | 0.03 |
| Pre-2014 Lifetime ND Symptoms | **-0.12** | **0.03** | **3.8E-06** | -0.16 | 0.10 | 0.09 |
| Pre-2014 Lifetime ASPD Symptoms | 0.01 | 0.03 | 0.8 | -0.15 | 0.08 | 0.07 |
| Adolescent Externalizing | **0.07** | **0.02** | **2.3E-06** | -0.42 | 0.16 | 0.01 |
| Parental Education | **-0.09** | **0.02** | **1.0E-06** | 0.12 | 0.14 | 0.39 |
| Sex | **0.22** | **0.02** | **1.3E-31** | 0.12 | 0.07 | 0.10 |
| Hispanic Ethnicity | **1.08** | **0.11** | **3.1E-22** | -1.07 | 0.34 | 1.8E-03 |
| Race | 0.38 | 0.26 | 0.14 | -0.60 | 2.03 | 0.77 |

Note: Bolded rows indicate significant estimates at the correct α=1.7×10^-3^ 

| Table S6: Co-Twin Control Results |  |  |  |  |  |  |  |  |  |  |  |  |
| --- | --- | --- | --- | --- | --- | --- | --- | --- | --- | --- | --- | --- |
| Phenotype | **Individual** |  |  | **MZ-DZ Combined Within-Pair** |  |  | **Same-Sex DZ** **Within-Pair** |  |  | **MZ Within-Pair** |  |  |
|  | **Beta** | **SE** | **P** | **Beta** | **SE** | **P** | **Beta** | **SE** | **P** | **Beta** | **SE** | **P** |
| Cannabis Frequency | **0.14** | **0.03** | **5.3E-06** | **0.11** | **0.03** | **1.3E-03** | 0.11 | 0.05 | 0.04 | 0.10 | 0.04 | 0.02 |
| Alcohol Frequency | -0.01 | 0.03 | 0.78 | 0.03 | 0.04 | 0.49 | 0.06 | 0.06 | 0.29 | -0.01 | 0.05 | 0.91 |
| Tobacco Frequency | **0.11** | **0.03** | **1.3E-04** | 0.00 | 0.03 | 0.95 | 0.03 | 0.05 | 0.53 | -0.03 | 0.04 | 0.49 |
| Other Illicit Drug Use | 0.06 | 0.04 | 0.08 | 0.06 | 0.04 | 0.14 | 0.10 | 0.07 | 0.15 | 0.03 | 0.05 | 0.59 |
| Cannabis Use Disorder | -0.05 | 0.03 | 0.17 | 0.04 | 0.04 | 0.29 | 0.04 | 0.06 | 0.54 | 0.05 | 0.05 | 0.35 |
| Alcohol Use Disorder | **-0.13** | **0.03** | **2.2E-04** | -0.11 | 0.04 | 6.7E-03 | -0.13 | 0.06 | 0.02 | -0.09 | 0.05 | 0.10 |
| Nicotine Dependence | -0.06 | 0.03 | 0.08 | -0.01 | 0.04 | 0.79 | 0.00 | 0.06 | 0.95 | -0.02 | 0.05 | 0.64 |
| Negative Affectivity | -0.07 | 0.04 | 0.04 | -0.05 | 0.04 | 0.18 | -0.02 | 0.06 | 0.71 | -0.07 | 0.05 | 0.13 |
| Detachment | -0.07 | 0.04 | 0.06 | -0.11 | 0.04 | 2.8E-03 | -0.15 | 0.06 | 0.01 | -0.08 | 0.05 | 0.11 |
| Psychoticism | 0.02 | 0.04 | 0.60 | -0.05 | 0.04 | 0.14 | -0.09 | 0.06 | 0.16 | -0.02 | 0.04 | 0.63 |
| Disinhibition | -0.09 | 0.03 | 0.01 | -0.07 | 0.04 | 0.07 | -0.17 | 0.06 | 0.01 | 0.02 | 0.05 | 0.61 |
| Current Externalizing | 0.03 | 0.04 | 0.47 | 0.02 | 0.04 | 0.66 | -0.04 | 0.06 | 0.55 | 0.07 | 0.05 | 0.20 |
| Savings Behavior | -0.10 | 0.03 | 3.1E-03 | 0.03 | 0.04 | 0.38 | 0.05 | 0.06 | 0.41 | 0.02 | 0.05 | 0.70 |
| Financial Distress | **0.13** | **0.04** | **4.4E-04** | -0.04 | 0.04 | 0.28 | -0.08 | 0.06 | 0.18 | 0.00 | 0.05 | 0.93 |
| Income | -0.05 | 0.03 | 0.12 | 0.02 | 0.03 | 0.60 | 0.03 | 0.06 | 0.62 | 0.01 | 0.04 | 0.85 |
| Unemployment | 0.10 | 0.04 | 3.9E-03 | 0.03 | 0.04 | 0.52 | 0.04 | 0.06 | 0.55 | 0.02 | 0.06 | 0.78 |
| Relationship Agreement | 0.07 | 0.04 | 0.09 | 0.02 | 0.05 | 0.61 | 0.02 | 0.07 | 0.78 | 0.03 | 0.06 | 0.62 |
| Legal Issues | 0.00 | 0.04 | 0.94 | -0.04 | 0.04 | 0.39 | -0.05 | 0.07 | 0.48 | -0.03 | 0.05 | 0.62 |
| Occupational Citizenship | -0.02 | 0.04 | 0.57 | -0.01 | 0.04 | 0.81 | -0.12 | 0.07 | 0.06 | 0.10 | 0.06 | 0.10 |
| Counter-productive Work | -0.05 | 0.04 | 0.16 | -0.08 | 0.04 | 0.06 | -0.11 | 0.06 | 0.09 | -0.05 | 0.05 | 0.37 |
| Community Attitude | -0.01 | 0.04 | 0.75 | -0.06 | 0.04 | 0.08 | -0.03 | 0.05 | 0.54 | -0.09 | 0.05 | 0.06 |
| Community Behavior | 0.04 | 0.04 | 0.26 | -0.02 | 0.04 | 0.61 | 0.00 | 0.06 | 0.99 | -0.03 | 0.05 | 0.46 |
| Cognitive Ability | 0.02 | 0.04 | 0.63 | 0.06 | 0.03 | 0.06 | 0.06 | 0.06 | 0.27 | 0.06 | 0.04 | 0.11 |

Note: Individual and combined MZ-DZ estimates presented in main text Figure 1.Bolded rows indicate significant

estimates at the correct α=2.3×10^-3^

| Table S7: Inverse Propensity Score Weighted Co-Twin Control Results for Sensitivity Analysis |  |  |  |  |  |  |  |  |  |  |  |  |
| --- | --- | --- | --- | --- | --- | --- | --- | --- | --- | --- | --- | --- |
| Phenotype | **Individual** |  |  | **MZ-DZ Combined Within-Pair** |  |  | **Same-Sex DZ** **Within-Pair** |  |  | **MZ Within-Pair** |  |  |
|  | **Beta** | **SE** | **P** | **Beta** | **SE** | **P** | **Beta** | **SE** | **P** | **Beta** | **SE** | **P** |
| Cannabis Frequency | **0.13** | **0.03** | **3.0E-05** | **0.11** | **0.03** | **1.8E-03** | 0.12 | 0.05 | 0.03 | 0.09 | 0.04 | 0.03 |
| Alcohol Frequency | -0.02 | 0.03 | 0.59 | 0.02 | 0.04 | 0.61 | 0.05 | 0.06 | 0.35 | -0.01 | 0.05 | 0.81 |
| Tobacco Frequency | **0.11** | **0.03** | **8.2E-05** | -0.01 | 0.03 | 0.82 | 0.02 | 0.05 | 0.65 | -0.03 | 0.04 | 0.46 |
| Other Illicit Drug Use | 0.07 | 0.04 | 0.08 | 0.06 | 0.04 | 0.14 | 0.11 | 0.07 | 0.13 | 0.02 | 0.05 | 0.67 |
| Cannabis Use Disorder | -0.08 | 0.03 | 0.03 | 0.02 | 0.04 | 0.69 | 0.04 | 0.06 | 0.50 | -0.01 | 0.05 | 0.91 |
| Alcohol Use Disorder | **-0.13** | **0.04** | **1.3E-04** | -0.11 | 0.04 | 6.9E-03 | -0.14 | 0.06 | 0.02 | -0.09 | 0.06 | 0.10 |
| Nicotine Dependence | -0.06 | 0.03 | 0.06 | -0.02 | 0.04 | 0.66 | -0.01 | 0.06 | 0.85 | -0.02 | 0.05 | 0.67 |
| Negative Affectivity | -0.07 | 0.04 | 0.07 | -0.05 | 0.04 | 0.21 | -0.01 | 0.06 | 0.84 | -0.08 | 0.05 | 0.10 |
| Detachment | -0.06 | 0.04 | 0.08 | -0.11 | 0.04 | 4.0E-03 | -0.14 | 0.06 | 0.03 | -0.08 | 0.05 | 0.09 |
| Psychoticism | 0.02 | 0.04 | 0.56 | -0.05 | 0.04 | 0.19 | -0.06 | 0.06 | 0.31 | -0.03 | 0.04 | 0.49 |
| Disinhibition | -0.09 | 0.04 | 8.5E-03 | -0.07 | 0.04 | 0.08 | -0.16 | 0.06 | 0.01 | 0.02 | 0.05 | 0.62 |
| Current Externalizing | 0.03 | 0.04 | 0.35 | 0.02 | 0.04 | 0.64 | -0.04 | 0.06 | 0.58 | 0.07 | 0.05 | 0.20 |
| Savings Behavior | -0.10 | 0.04 | 4.0E-03 | 0.03 | 0.04 | 0.35 | 0.04 | 0.06 | 0.52 | 0.03 | 0.05 | 0.52 |
| Financial Distress | **0.13** | **0.04** | **1.2E-03** | -0.05 | 0.04 | 0.25 | -0.07 | 0.06 | 0.25 | -0.02 | 0.05 | 0.69 |
| Income | -0.05 | 0.03 | 0.12 | 0.03 | 0.04 | 0.45 | 0.04 | 0.06 | 0.46 | 0.01 | 0.04 | 0.81 |
| Unemployment | 0.09 | 0.04 | 0.01 | 0.01 | 0.04 | 0.74 | 0.02 | 0.06 | 0.70 | 0.00 | 0.06 | 0.94 |
| Relationship Agreement | 0.07 | 0.04 | 0.09 | 0.03 | 0.05 | 0.53 | 0.05 | 0.07 | 0.49 | 0.01 | 0.06 | 0.83 |
| Legal Issues | 0.00 | 0.04 | 0.94 | -0.03 | 0.04 | 0.54 | -0.02 | 0.07 | 0.73 | -0.03 | 0.05 | 0.60 |
| Occupational Citizenship | -0.02 | 0.04 | 0.61 | -0.01 | 0.04 | 0.78 | -0.12 | 0.07 | 0.07 | 0.09 | 0.06 | 0.13 |
| Counter-productive Work | -0.04 | 0.04 | 0.25 | -0.05 | 0.04 | 0.23 | -0.06 | 0.06 | 0.31 | -0.03 | 0.05 | 0.53 |
| Community Attitude | -0.02 | 0.04 | 0.60 | -0.07 | 0.04 | 0.06 | -0.05 | 0.06 | 0.36 | -0.09 | 0.05 | 0.08 |
| Community Behavior | 0.04 | 0.04 | 0.25 | -0.02 | 0.04 | 0.65 | -0.01 | 0.06 | 0.82 | -0.02 | 0.05 | 0.66 |
| Cognitive Ability | 0.02 | 0.04 | 0.53 | 0.06 | 0.03 | 0.06 | 0.06 | 0.06 | 0.26 | 0.06 | 0.04 | 0.12 |

Note: Bolded rows indicate significant estimates at the correct α=2.3×10^-3^

| Table S8: Sensitivity Analysis of Recreational Environment Definition |  |  |  |  |  |  |  |  |  |
| --- | --- | --- | --- | --- | --- | --- | --- | --- | --- |
| Phenotype | **Individual** |  |  | **MZ-DZ Combined Co-Twin Control** |  |  |  |  |  |
|  |  |  |  | **Between-Pair** |  |  | **Within-Pair** |  |  |
|  | **Beta** | **SE** | **P** | **Beta** | **SE** | **P** | **Beta** | **SE** | **P** |
| Cannabis Frequency | **0.15** | **0.03** | **1.6E-06** | 0.10 | 0.04 | 0.01 | **0.15** | **0.03** | **1.5E-05** |
| Alcohol Frequency | -0.04 | 0.03 | 0.22 | -0.06 | 0.04 | 0.16 | 0.00 | 0.04 | 0.97 |
| Tobacco Frequency | **0.12** | **0.03** | **2.7E-05** | **0.15** | **0.04** | **4.7E-05** | 0.01 | 0.03 | 0.74 |
| Other Illicit Drug Use | 0.06 | 0.04 | 0.07 | 0.04 | 0.05 | 0.36 | 0.06 | 0.04 | 0.15 |
| Cannabis Use Disorder | -0.05 | 0.03 | 0.16 | -0.06 | 0.04 | 0.19 | 0.05 | 0.04 | 0.23 |
| Alcohol Use Disorder | **-0.13** | **0.03** | **1.5E-04** | -0.13 | 0.04 | 2.4E-03 | -0.12 | 0.04 | 2.5E-03 |
| Nicotine Dependence | -0.07 | 0.03 | 0.04 | -0.09 | 0.04 | 0.02 | -0.01 | 0.04 | 0.84 |
| Negative Affectivity | -0.08 | 0.04 | 0.04 | -0.02 | 0.05 | 0.68 | -0.04 | 0.04 | 0.26 |
| Detachment | -0.07 | 0.04 | 0.04 | 0.03 | 0.05 | 0.58 | **-0.14** | **0.04** | **5.4E-04** |
| Psychoticism | 0.03 | 0.04 | 0.33 | 0.12 | 0.05 | 0.01 | -0.05 | 0.04 | 0.17 |
| Disinhibition | -0.08 | 0.04 | 0.02 | -0.05 | 0.05 | 0.32 | -0.07 | 0.04 | 0.08 |
| Current Externalizing | 0.03 | 0.04 | 0.46 | 0.05 | 0.04 | 0.30 | 0.01 | 0.04 | 0.89 |
| Savings Behavior | -0.09 | 0.04 | 0.01 | **-0.18** | **0.05** | **1.4E-04** | 0.07 | 0.04 | 0.06 |
| Financial Distress | **0.14** | **0.04** | **2.7E-04** | **0.23** | **0.05** | **9.4E-06** | -0.04 | 0.04 | 0.28 |
| Income | -0.07 | 0.03 | 0.06 | -0.10 | 0.05 | 0.02 | 0.03 | 0.04 | 0.40 |
| Unemployment | 0.08 | 0.04 | 0.03 | 0.08 | 0.05 | 0.07 | -0.04 | 0.04 | 0.32 |
| Relationship Agreement | 0.08 | 0.04 | 0.05 | 0.07 | 0.05 | 0.18 | 0.07 | 0.05 | 0.12 |
| Legal Issues | 0.00 | 0.04 | 0.99 | 0.00 | 0.04 | 0.97 | -0.06 | 0.04 | 0.21 |
| Occupational Citizenship | -0.03 | 0.04 | 0.41 | -0.07 | 0.05 | 0.16 | -0.01 | 0.05 | 0.82 |
| Counterproductive Work Behavior | -0.06 | 0.04 | 0.11 | 0.00 | 0.05 | 0.93 | -0.06 | 0.04 | 0.18 |
| Community Attitude | -0.02 | 0.04 | 0.61 | -0.02 | 0.05 | 0.64 | -0.04 | 0.04 | 0.31 |
| Community Behavior | 0.04 | 0.04 | 0.29 | 0.01 | 0.05 | 0.91 | 0.02 | 0.04 | 0.63 |
| Cognitive Ability | -0.03 | 0.04 | 0.48 | -0.09 | 0.05 | 0.06 | 0.05 | 0.03 | 0.12 |

Note: No broad differences identified between these sensitivity analyses and primary analyses. Bolded rows indicate significant estimates at the correct α=2.3×10^-3^ 

| Table S9: Sensitivity Analyses of Ordinal Frequency and Binary Diagnosis |  |  |  |  |  |  |  |  |  |
| --- | --- | --- | --- | --- | --- | --- | --- | --- | --- |
|  | **Individual** |  |  | **MZ-DZ Combined Co-Twin Control** |  |  |  |  |  |
|  |  |  |  | **Between-Pair** |  |  | **Within-Pair** |  |  |
| Phenotype | **Beta** | **SE** | **P** | **Beta** | **SE** | **P** | **Beta** | **SE** | **P** |
| Cannabis Frequency | **0.26** | **0.06** | **1.7E-05** | 0.22 | 0.08 | 4.5E-03 | **0.23** | **0.07** | **4.7E-04** |
| Alcohol Frequency | -0.09 | 0.06 | 0.17 | -0.13 | 0.08 | 0.11 | -0.01 | 0.07 | 0.87 |
| Cannabis Use Disorder | 0.10 | 0.17 | 0.56 | 0.14 | 0.22 | 0.53 | 0.36 | 0.21 | 0.09 |
| Alcohol Use Disorder | **-0.35** | **0.11** | **1.9E-03** | -0.17 | 0.14 | 0.24 | **-0.48** | **0.14** | **5.5E-04** |
| Nicotine Dependence | -0.25 | 0.13 | 0.06 | -0.25 | 0.17 | 0.14 | -0.11 | 0.17 | 0.54 |

Note: No broad differences identified between these sensitivity analyses and primary analyses. Bolded rows 

indicate significant estimates at the correct α=2.3×10^-3^ 

| Table S10: Sensitivity Analysis of Covariates |  |  |  |  |  |  |  |  |  |
| --- | --- | --- | --- | --- | --- | --- | --- | --- | --- |
| Phenotype | **Individual** |  |  | **MZ-DZ Combined Co-Twin Control** |  |  |  |  |  |
|  |  |  |  | **Between-Pair** |  |  | **Within-Pair** |  |  |
|  | **Beta** | **SE** | **P** | **Beta** | **SE** | **P** | **Beta** | **SE** | **P** |
| Removal of Pre-Legalization Covariate |  |  |  |  |  |  |  |  |  |
| Cannabis Frequency | **0.13** | **0.04** | **2.4E-04** | 0.11 | 0.05 | 0.01 | 0.08 | 0.04 | 0.02 |
| Alcohol Frequency | -0.04 | 0.04 | 0.32 | -0.04 | 0.05 | 0.45 | -0.04 | 0.04 | 0.31 |
| Tobacco Frequency | -0.02 | 0.04 | 0.67 | 0.00 | 0.05 | 0.99 | -0.03 | 0.04 | 0.42 |
| Cannabis Use Disorder | -0.02 | 0.04 | 0.61 | -0.01 | 0.05 | 0.81 | 0.04 | 0.04 | 0.33 |
| Alcohol Use Disorder | -0.06 | 0.04 | 0.11 | -0.01 | 0.04 | 0.88 | -0.12 | 0.04 | 0.00 |
| Nicotine Dependence | -0.03 | 0.04 | 0.45 | -0.04 | 0.04 | 0.42 | -0.03 | 0.04 | 0.48 |
| Disinhibition | -0.05 | 0.04 | 0.15 | -0.02 | 0.05 | 0.62 | -0.06 | 0.04 | 0.10 |
| Current Externalizing | 0.04 | 0.03 | 0.23 | 0.06 | 0.04 | 0.17 | 0.02 | 0.04 | 0.62 |
| Savings Behavior | -0.06 | 0.04 | 0.07 | -0.13 | 0.05 | 5.1E-03 | 0.03 | 0.04 | 0.41 |
| Financial Distress | 0.10 | 0.04 | 0.01 | **0.19** | **0.05** | **2.3E-04** | -0.04 | 0.04 | 0.26 |
| Income | -0.01 | 0.03 | 0.70 | -0.06 | 0.05 | 0.16 | 0.02 | 0.03 | 0.56 |
| Unemployment | 0.09 | 0.04 | 0.01 | 0.08 | 0.04 | 0.07 | 0.03 | 0.04 | 0.49 |
| Legal Issues | 0.01 | 0.04 | 0.77 | 0.01 | 0.04 | 0.87 | -0.05 | 0.04 | 0.26 |
| Adding Covariate of Pre-Legalization Assessment Year |  |  |  |  |  |  |  |  |  |
| Cannabis Frequency | **0.23** | **0.05** | **3.2E-06** | 0.18 | 0.06 | 3.1E-03 | **0.17** | **0.05** | **1.3E-03** |
| Alcohol Frequency | -0.04 | 0.05 | 0.45 | -0.10 | 0.07 | 0.17 | 0.04 | 0.06 | 0.50 |
| Tobacco Frequency | 0.13 | 0.05 | 0.02 | 0.18 | 0.07 | 0.01 | -0.01 | 0.06 | 0.93 |

 Note: No broad differences identified between these sensitivity analyses and primary analyses. Bolded rows indicate significant estimates at the correct α=2.3×10^-3^ 

| Table S11: Results of Covid Sensitivity Analyses on Financial Outcomes |  |  |  |  |  |  |  |  |  |
| --- | --- | --- | --- | --- | --- | --- | --- | --- | --- |
|  | **Individual** |  |  |  |  |  |  |  |  |
|  | **Legalization** |  |  | **Covid** |  |  |  |  |  |
|  | **Beta** | **SE** | **P** | **Beta** |  | **SE** |  | **P** |  |
| Savings Behavior | -0.10 | 0.03 | 3.5E-03 | 0.06 |  | 0.03 |  | 0.08 |  |
| Financial Distress | **0.13** | **0.04** | **3.9E-04** | **-0.18** |  | **0.04** |  | **7.4E-06** |  |
| Income | -0.05 | 0.03 | 0.13 | **0.12** |  | **0.03** |  | **6.1E-04** |  |
| Unemployment | 0.11 | 0.04 | 2.7E-03 | **0.13** |  | **0.03** |  | **1.1E-04** |  |
|  | **MZ-DZ Combined Co-Twin** |  |  |  |  |  |  |  |  |
|  | **Legalization** **Between-Pair** |  |  | **Legalization** **Within-Pair** |  |  | **Covid** |  |  |
|  | **Beta** | **SE** | **P** | **Beta** | **SE** | **P** | **Beta** | **SE** | **P** |
| Savings Behavior | **-0.18** | **0.05** | **1.2E-04** | 0.03 | 0.04 | 0.38 | 0.06 | 0.04 | -0.18 |
| Financial Distress | **0.22** | **0.05** | **1.5E-05** | -0.04 | 0.04 | 0.29 | -0.19 | 0.05 | 0.22 |
| Income | -0.10 | 0.04 | 0.02 | 0.02 | 0.03 | 0.61 | 0.11 | 0.04 | -0.10 |
| Unemployment | 0.11 | 0.04 | 0.02 | 0.03 | 0.04 | 0.53 | 0.14 | 0.04 | 0.11 |

Note: No broad differences identified between these sensitivity analyses and primary analyses. Bolded rows 

indicate significant estimates at the correct α=2.3×10^-3^

| Table S12: Differential Vulnerability Analyses |  |  |  |  |  |  |  |  |  |  |  |  |
| --- | --- | --- | --- | --- | --- | --- | --- | --- | --- | --- | --- | --- |
| Phenotype | **Age** |  |  |  |  |  | **Sex** |  |  |  |  |  |
|  | **Main Effect** |  |  | **Interaction** |  |  | **Main Effect** |  |  | **Interaction** |  |  |
|  | **Beta** | **SE** | **p-value** | **Beta** | **SE** | **p-value** | **Beta** | **SE** | **p-value** | **Beta** | **SE** | **p-value** |
| Cannabis Frequency | -0.122 | 0.039 | 1.83E-03 | -0.047 | 0.058 | 0.42 | **-0.018** | **0.004** | **1.11E-06** | 0.003 | 0.008 | 0.74 |
| Alcohol Frequency | -0.140 | 0.042 | 8.44E-04 | 0.114 | 0.063 | 0.07 | -0.014 | 0.004 | 5.81E-04 | 0.024 | 0.008 | 0.00 |
| Tobacco Frequency | **-0.199** | **0.036** | **2.78E-08** | 0.044 | 0.054 | 0.41 | **-0.018** | **0.003** | **1.16E-07** | 0.007 | 0.007 | 0.33 |
| Other Illicit Drug Use | **-0.177** | **0.044** | **7.28E-05** | -0.072 | 0.068 | 0.29 | **-0.023** | **0.004** | **4.36E-08** | -0.004 | 0.009 | 0.62 |
| Cannabis Use Disorder | -0.142 | 0.042 | 6.95E-04 | 0.023 | 0.064 | 0.72 | **-0.021** | **0.004** | **1.60E-07** | -0.012 | 0.008 | 0.15 |
| Alcohol Use Disorder | **-0.164** | **0.043** | **1.20E-04** | 0.036 | 0.065 | 0.58 | **-0.020** | **0.004** | **4.09E-07** | 0.005 | 0.008 | 0.51 |
| Nicotine Dependence | **-0.201** | **0.039** | **3.55E-07** | 0.085 | 0.061 | 0.17 | **-0.019** | **0.004** | **4.02E-07** | -0.003 | 0.008 | 0.66 |
| Negative Affectivity | **0.233** | **0.045** | **2.68E-07** | 0.072 | 0.067 | 0.28 | **-0.021** | **0.004** | **8.89E-07** | -0.001 | 0.009 | 0.94 |
| Detachment | **-0.202** | **0.046** | **9.94E-06** | 0.017 | 0.068 | 0.80 | -0.009 | 0.004 | 3.36E-02 | -0.001 | 0.009 | 0.92 |
| Psychoticism | **-0.331** | **0.045** | **2.59E-13** | -0.020 | 0.067 | 0.76 | **-0.021** | **0.004** | **1.15E-06** | -0.014 | 0.009 | 0.11 |
| Disinhibition | **-0.263** | **0.044** | **2.96E-09** | -0.059 | 0.066 | 0.37 | **-0.023** | **0.004** | **3.94E-08** | 0.004 | 0.009 | 0.60 |
| Current Externalizing | -0.128 | 0.044 | 3.61E-03 | 0.059 | 0.067 | 0.38 | -0.013 | 0.004 | 1.71E-03 | -0.003 | 0.009 | 0.72 |
| Savings Behavior | 0.004 | 0.044 | 0.93 | 0.035 | 0.066 | 0.59 | **0.042** | **0.004** | **3.92E-22** | 0.000 | 0.008 | 0.99 |
| Financial Distress | 0.122 | 0.052 | 0.02 | 0.032 | 0.072 | 0.66 | **-0.022** | **0.005** | **6.49E-06** | 0.004 | 0.009 | 0.70 |
| Income | **-0.457** | **0.043** | **9.33E-26** | 0.005 | 0.064 | 0.94 | **0.048** | **0.004** | **6.53E-29** | 0.000 | 0.008 | 0.97 |
| Unemployment | 0.040 | 0.044 | 0.36 | 0.034 | 0.068 | 0.62 | -0.013 | 0.004 | 1.95E-03 | 0.028 | 0.009 | 0.00 |
| Relationship Agreement | 0.080 | 0.049 | 0.11 | 0.015 | 0.076 | 0.84 | **-0.020** | **0.005** | **1.52E-05** | 0.002 | 0.010 | 0.87 |
| Legal Issues | -0.008 | 0.044 | 0.86 | -0.061 | 0.068 | 0.37 | **-0.017** | **0.004** | **3.61E-05** | -0.001 | 0.009 | 0.88 |
| Occupational Citizenship | 0.042 | 0.046 | 0.35 | -0.064 | 0.070 | 0.37 | -0.002 | 0.004 | 0.61 | 0.011 | 0.009 | 0.21 |
| Counterproductive Work Behavior | **-0.223** | **0.046** | **1.54E-06** | 0.060 | 0.070 | 0.39 | **-0.020** | **0.004** | **4.32E-06** | -0.005 | 0.009 | 0.58 |
| Community Attitude | **0.168** | **0.046** | **2.87E-04** | -0.093 | 0.068 | 0.17 | 0.005 | 0.004 | 0.21 | 0.010 | 0.009 | 0.26 |
| Community Behavior | **0.162** | **0.046** | **4.26E-04** | -0.101 | 0.067 | 0.13 | **0.024** | **0.004** | **4.12E-08** | 0.012 | 0.009 | 0.17 |
| Cognitive Ability | **-0.208** | **0.047** | **9.01E-06** | -0.031 | 0.067 | 0.65 | -0.013 | 0.004 | 3.54E-03 | 0.014 | 0.009 | 0.11 |
| Phenotype | **Adolescent Externalizing** |  |  |  |  |  | **Adult Antisocial Personality Disorder** |  |  |  |  |  |
|  | **Main Effect** |  |  | **Interaction** |  |  | **Main Effect** |  |  | **Interaction** |  |  |
|  | **Beta** | **SE** | **P** | **Beta** | **SE** | **P** | **Beta** | **SE** | **P** | **Beta** | **SE** | **P** |
| Cannabis Frequency | **0.030** | **0.008** | **1.95E-04** | 0.004 | 0.012 | 0.74 | 0.038 | 0.014 | 7.16E-03 | 0.018 | 0.018 | 0.32 |
| Alcohol Frequency | -0.011 | 0.009 | 0.23 | -0.005 | 0.013 | 0.70 | **-0.066** | **0.015** | **1.21E-05** | 0.016 | 0.020 | 0.42 |
| Tobacco Frequency | **0.033** | **0.008** | **1.39E-05** | -0.008 | 0.011 | 0.44 | **0.076** | **0.013** | **6.09E-09** | -0.017 | 0.017 | 0.32 |
| Other Illicit Drug Use | 0.029 | 0.009 | 1.94E-03 | 0.002 | 0.014 | 0.87 | **0.067** | **0.016** | **2.63E-05** | 0.026 | 0.021 | 0.22 |
| Cannabis Use Disorder | **0.038** | **0.009** | **1.86E-05** | -0.032 | 0.013 | 0.01 | 0.005 | 0.016 | 0.77 | 0.026 | 0.020 | 0.20 |
| Alcohol Use Disorder | 0.008 | 0.009 | 0.35 | -0.007 | 0.013 | 0.59 | 0.016 | 0.017 | 0.34 | -0.007 | 0.020 | 0.75 |
| Nicotine Dependence | 0.008 | 0.009 | 0.32 | -0.012 | 0.012 | 0.35 | 0.043 | 0.016 | 5.37E-03 | -0.026 | 0.019 | 0.17 |
| Negative Affectivity | 0.025 | 0.009 | 6.21E-03 | 0.000 | 0.013 | 0.98 | **0.068** | **0.016** | **1.92E-05** | -0.005 | 0.021 | 0.79 |
| Detachment | **0.051** | **0.009** | **3.61E-08** | -0.018 | 0.013 | 0.18 | **0.138** | **0.016** | **7.27E-18** | -0.054 | 0.021 | 0.01 |
| Psychoticism | **0.065** | **0.009** | **1.26E-12** | -0.006 | 0.013 | 0.64 | **0.147** | **0.016** | **6.20E-21** | -0.048 | 0.020 | 0.02 |
| Disinhibition | **0.087** | **0.009** | **9.39E-22** | -0.010 | 0.013 | 0.45 | **0.138** | **0.016** | **1.62E-18** | -0.035 | 0.020 | 0.08 |
| Current Externalizing | **0.052** | **0.009** | **2.94E-08** | -0.005 | 0.014 | 0.69 | **0.064** | **0.016** | **8.36E-05** | 0.043 | 0.021 | 0.04 |
| Savings Behavior | **-0.038** | **0.009** | **1.85E-05** | -0.022 | 0.013 | 0.09 | **-0.125** | **0.015** | **3.31E-16** | -0.011 | 0.020 | 0.58 |
| Financial Distress | **0.062** | **0.011** | **8.75E-09** | -0.007 | 0.014 | 0.62 | **0.148** | **0.019** | **8.21E-15** | -0.015 | 0.023 | 0.50 |
| Income | -0.028 | 0.009 | 1.15E-03 | 0.009 | 0.013 | 0.49 | **-0.085** | **0.015** | **1.46E-08** | 0.018 | 0.020 | 0.36 |
| Unemployment | 0.015 | 0.009 | 0.10 | 0.006 | 0.014 | 0.66 | **0.099** | **0.016** | **7.82E-10** | -0.028 | 0.021 | 0.18 |
| Relationship Agreement | **-0.042** | **0.011** | **1.29E-04** | 0.003 | 0.016 | 0.84 | **-0.113** | **0.018** | **6.73E-10** | 0.029 | 0.024 | 0.23 |
| Legal Issues | **0.041** | **0.009** | **1.43E-05** | 0.007 | 0.014 | 0.60 | **0.098** | **0.016** | **2.00E-09** | -0.024 | 0.021 | 0.26 |
| Occupational Citizenship | 0.026 | 0.010 | 9.33E-03 | -0.015 | 0.014 | 0.31 | 0.013 | 0.017 | 0.43 | -0.015 | 0.022 | 0.51 |
| Counter-productive Work Behavior | **0.058** | **0.010** | **2.49E-09** | -0.032 | 0.014 | 0.02 | **0.123** | **0.017** | **1.20E-13** | -0.047 | 0.022 | 0.03 |
| Community Attitude | **-0.039** | **0.009** | **2.26E-05** | 0.012 | 0.013 | 0.37 | **-0.077** | **0.016** | **1.37E-06** | 0.009 | 0.021 | 0.66 |
| Community Behavior | -0.029 | 0.009 | 1.24E-03 | 0.009 | 0.013 | 0.52 | **-0.074** | **0.016** | **2.74E-06** | 0.014 | 0.020 | 0.49 |
| Cognitive Ability | **-0.042** | **0.009** | **2.21E-06** | 0.005 | 0.013 | 0.69 | **-0.094** | **0.015** | **1.17E-09** | 0.028 | 0.020 | 0.16 |

Note: Main Text figure 2 depicts these estimates. Bolded rows indicate significant estimates at the correct α=4.6×10^-4^

| Table S13: Power to Detect Interaction Effects in Differential Vulnerability Analyses |  |  |  |  |  |  |  |  |
| --- | --- | --- | --- | --- | --- | --- | --- | --- |
|  | **Age** |  | **Sex** |  | **Adolescent Externalizing** |  | **Adult ASPD** |  |
| Phenotype | **Sample Size** | **Power** | **Sample Size** | **Power** | **Sample Size** | **Power** | **Sample Size** | **Power** |
| Cannabis Frequency | **3805** | **0.96** | 3805 | 0.44 | **3708** | **1.00** | **3805** | **1.00** |
| Alcohol Frequency | 3877 | 0.15 | 3877 | 0.60 | 3779 | 0.24 | 3877 | 0.04 |
| Tobacco Frequency | 3871 | 0.33 | **3871** | **0.95** | **3772** | **1.00** | **3871** | **1.00** |
| Other Illicit Drug Use | 3921 | 0.60 | 3921 | 0.14 | **3815** | **0.98** | **3920** | **1.00** |
| Cannabis Use Disorder | 3890 | 0.14 | 3890 | 0.32 | **3785** | **0.85** | **3890** | **0.99** |
| Alcohol Use Disorder | 3903 | 0.28 | **3903** | **0.83** | 3798 | 0.58 | **3903** | **0.99** |
| Nicotine Dependence | 3905 | 0.33 | 3905 | 0.76 | **3800** | **0.98** | **3905** | **1.00** |
| Negative Affectivity | 3908 | 0.27 | 3908 | 0.23 | 3801 | 0.35 | 3907 | 0.27 |
| Detachment | 3907 | 0.23 | 3907 | 0.53 | **3800** | **0.98** | **3906** | **0.99** |
| Psychoticism | 3906 | 0.11 | 3906 | 0.23 | **3799** | **1.00** | **3905** | **1.00** |
| Disinhibition | 3800 | 0.42 | **3800** | **0.99** | **3800** | **1.00** | **3906** | **1.00** |
| Current Externalizing | 3818 | 0.10 | 3818 | 0.09 | **3818** | **1.00** | **3924** | **1.00** |
| Savings Behavior | 3892 | 0.76 | **3892** | **0.81** | **3785** | **1.00** | **3892** | **1.00** |
| Financial Distress | 3172 | 0.68 | **3172** | **0.96** | **3069** | **1.00** | **3172** | **1.00** |
| Income | 3871 | 0.33 | **3871** | **1.00** | 3764 | 0.74 | **3871** | **0.96** |
| Unemployment | **3915** | **0.80** | **3915** | **0.90** | **3808** | **0.87** | **3915** | **1.00** |
| Relationship Agreement | 3108 | 0.59 | **3108** | **0.88** | 3024 | 0.70 | **3107** | **0.93** |
| Legal Issues | 3818 | 0.08 | 3818 | 0.07 | **3818** | **1.00** | **3925** | **1.00** |
| Occupational Citizenship | 3623 | 0.06 | 3623 | 0.08 | 3526 | 0.07 | 3623 | 0.05 |
| Counterproductive Work Behavior | 3622 | 0.13 | 3622 | 0.25 | 3525 | 0.68 | **3622** | **0.95** |
| Community Attitude | 3923 | 0.08 | 3923 | 0.07 | **3816** | **0.87** | **3922** | **0.99** |
| Community Behavior | 3924 | 0.22 | 3924 | 0.19 | 3817 | 0.69 | **3923** | **0.91** |
| Cognitive Ability | 3920 | 0.15 | 3920 | 0.23 | **3812** | **0.90** | **3919** | **0.91** |

Note: Bolding denotes power equal to or greater than 0.80 to detect an interaction effect. 

**Figure S1.** Histograms of test years pre-legalization (top left), ages pre-legalization (bottom left), test years post-legalization (top right), and ages post-legalization (bottom right). 

 

**Figure S2.** Heat map of individual level correlations between continuous variables. Note: Freq. = Frequency; CUD = Cannabis Use Disorder; AUD = Alcohol Use Disorder; ND = Nicotine Dependence; ASPD = Antisocial Personality Disorder.
